# The effects of implementing a point-of-care electronic template to prompt routine anxiety and depression screening in patients consulting for osteoarthritis (the Primary Care Osteoarthritis Trial): A cluster randomised trial in primary care

**DOI:** 10.1371/journal.pmed.1002273

**Published:** 2017-04-11

**Authors:** Christian D. Mallen, Barbara I. Nicholl, Martyn Lewis, Bernadette Bartlam, Daniel Green, Sue Jowett, Jesse Kigozi, John Belcher, Kris Clarkson, Zoe Lingard, Christopher Pope, Carolyn A. Chew-Graham, Peter Croft, Elaine M. Hay, George Peat

**Affiliations:** 1 Arthritis Research UK Primary Care Centre, Research Institute for Primary Care and Health Sciences, Keele University, Keele, United Kingdom; 2 NIHR Collaboration for Leadership in Applied Health Research and Care West Midlands, Keele, United Kingdom; 3 General Practice and Primary Care, Institute of Health and Wellbeing, University of Glasgow, Glasgow, United Kingdom; Massachusetts General Hospital, UNITED STATES

## Abstract

**Background:**

This study aimed to evaluate whether prompting general practitioners (GPs) to routinely assess and manage anxiety and depression in patients consulting with osteoarthritis (OA) improves pain outcomes.

**Methods and findings:**

We conducted a cluster randomised controlled trial involving 45 English general practices. In intervention practices, patients aged ≥45 y consulting with OA received point-of-care anxiety and depression screening by the GP, prompted by an automated electronic template comprising five questions (a two-item Patient Health Questionnaire–2 for depression, a two-item Generalized Anxiety Disorder–2 questionnaire for anxiety, and a question about current pain intensity [0–10 numerical rating scale]). The template signposted GPs to follow National Institute for Health and Care Excellence clinical guidelines for anxiety, depression, and OA and was supported by a brief training package. The template in control practices prompted GPs to ask the pain intensity question only. The primary outcome was patient-reported current pain intensity post-consultation and at 3-, 6-, and 12-mo follow-up. Secondary outcomes included pain-related disability, anxiety, depression, and general health.

During the trial period, 7,279 patients aged ≥45 y consulted with a relevant OA-related code, and 4,240 patients were deemed potentially eligible by participating GPs. Templates were completed for 2,042 patients (1,339 [31.6%] in the control arm and 703 [23.1%] in the intervention arm). Of these 2,042 patients, 1,412 returned questionnaires (501 [71.3%] from 20 intervention practices, 911 [68.0%] from 24 control practices). Follow-up rates were similar in both arms, totalling 1,093 (77.4%) at 3 mo, 1,064 (75.4%) at 6 mo, and 1,017 (72.0%) at 12 mo. For the primary endpoint, multilevel modelling yielded significantly higher average pain intensity across follow-up to 12 mo in the intervention group than the control group (adjusted mean difference 0.31; 95% CI 0.04, 0.59). Secondary outcomes were consistent with the primary outcome measure in reflecting better outcomes as a whole for the control group than the intervention group. Anxiety and depression scores did not reduce following the intervention. The main limitations of this study are two potential sources of bias: an imbalance in cluster size (mean practice size 7,397 [intervention] versus 5,850 [control]) and a difference in the proportion of patients for whom the GP deactivated the template (33.6% [intervention] versus 27.8% [control]).

**Conclusions:**

In this study, we observed no beneficial effect on pain outcomes of prompting GPs to routinely screen for and manage comorbid anxiety and depression in patients presenting with symptoms due to OA, with those in the intervention group reporting statistically significantly higher average pain scores over the four follow-up time points than those in the control group.

**Trial registration:**

ISRCTN registry ISRCTN40721988

## Abstract 2

Christian Mallen and colleagues report on a cluster-randomized trial investigating screening for anxiety and depression in patients presenting with osteoarthritis.

## Author summary

**Why was this study done?:**

- Patients with painful conditions, such as osteoarthritis, are at higher risk of experiencing depression and anxiety.
- People with pain and coexisting mood problems are more likely to have a poorer long-term outcome.
- Routine screening for anxiety and depression in higher risk patients is controversial, with conflicting evidence.

**What did the researchers do and find?:**

- We conducted a large cluster randomised trial to investigate the impact on pain of screening and treating people with osteoarthritis for depression and anxiety in the general practice consultation.
- General practices were randomised either to routinely screen patients consulting with osteoarthritis for depression and anxiety or to not screen.
- We found that those screened for depression and anxiety had worse pain outcomes over the following 12 months than those not screened.

**What do these findings mean?:**

- Our findings do not support the implementation of routine screening of patients with osteoarthritis for depression and anxiety in primary care.
- Further work is needed to determine the potential benefit of screening patients with different musculoskeletal disorders.

## Introduction

Osteoarthritis (OA) is a major cause of persistent pain and years lived with disability [1], and one of the most common reasons for primary care consultation in the UK, with approximately 1 million adults seeking care each year [2]. Patients with painful, disabling OA constitute a high-risk group for distress, anxiety, and depressive disorders [3–5]. While many of the factors associated with the future course of OA are not modifiable (e.g., age, sex, symptom duration, and severity of underlying structural changes to the joint [6–9]), comorbid depression and anxiety are adversely related to future course [10,11], treatment response [12], and healthcare use [13], and show a reciprocal relationship with pain and functional outcomes [12,14,15]. Evidence from a single clinical trial of a collaborative care approach using psychological therapies and medication management for previously diagnosed major depressive disorder in patients with self-reported OA supports the principle of managing comorbid depression as a means of modifying general and OA-specific clinical outcomes, having shown beneficial effects of depression management on pain intensity, pain-related function, and quality of life sustained to 12 mo [16–18]. Similar benefits may accrue from the effective management of comorbid anxiety disorders in patients with persistent painful disorders [19,20].

Yet the proposal of screening for depression in this and other high-risk groups is contentious, despite some evidence that depression is under-recognised in patients presenting to primary care with painful OA [12,21,22]. Screening for anxiety in primary care has been occasionally proposed [23–25], but to our knowledge has not been evaluated in clinical trials in UK primary care. With respect to depression, in both unselected primary care populations and special populations at high risk of depression, several recent systematic reviews by Thombs and colleagues [26–28] and others [29] have highlighted a lack of direct evidence from appropriately designed clinical trials on the effects of implementing screening for depression, either alone or in the context of accessible, good-quality mental healthcare.

The UK National Institute for Health and Care Excellence (NICE) guidelines for OA are ambiguous on the matter of screening for depression and anxiety, recommending that patients be assessed for the effect of OA on mood, and specifically including “screen for depression” as a topic “worth assessing”, but acknowledging that it may not be of concern for every patient [30]. Similarly, NICE guideline 91 [31] suggests practitioners should be alert to depression in patients with chronic physical problems and should consider asking two short case-finding questions [32] of patients whom they suspect of having depression. The potential benefits of screening in OA may not just be about the screen–diagnose–treat pathway for patients with anxiety or depression. The recognition of sub-threshold anxiety and depression symptoms—more common and still associated with poorer pain and function outcomes—could “open the door to a dialogue with clinicians who can then determine which unmet needs have contributed to distress” [33]. This could include exploring causes (e.g., poorly controlled pain [34], sleep disturbance [35], or inadequate social support [36]) as well as prompting greater use of pain management strategies and functional rehabilitation options, such as referral to physiotherapy for supervised exercise that is effective for pain [37], function [37], and mental health [38,39] but typically underutilised [40,41]. Against these potential benefits can be raised a number of concerns about a systematic approach to screening [28,42–46]: inefficiency, diversion of scarce resources, potential for unnecessary exposure to antidepressant medication side effects, possible nocebo effects, stigma from overt labelling, “psychologising” of the pain problem, and mechanical delivery.

In this study our primary objective was to evaluate the clinical effectiveness of introducing general practitioner (GP) screening for anxiety and depression in older patients consulting for OA. Specifically, we hypothesized that patients undergoing screening for anxiety and depression symptoms in the GP consultation would show greater improvements in current pain intensity and pain interference with daily activity over the 12 mo following their consultation, compared to those having a standard GP consultation.

## Methods

### Trial design

**Fig 1:**
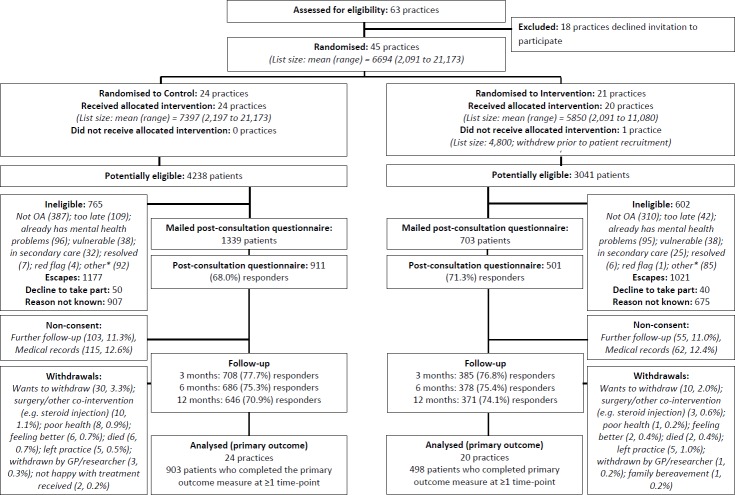
Participant flow. *The category “other” for ineligibility includes the following: not a GP appointment (administrative/nurse), telephone contact, already in another study, received injection, moving practice/house, working away, and difficulty understanding/communicating in English. GP, general practitioner; OA, osteoarthritis.

This was a pragmatic cluster randomised parallel trial in primary care. Randomisation of general practices (rather than individual GPs or individual patients) was chosen because we anticipated GPs would likely find it difficult to ask screening questions of some patients and not others allocated at random, and therefore the potential for contamination between the two arms, as well as between GPs within a practice allocated to different arms, was considered highly likely [47]. Clusters were general practices that were randomly assigned (1:1) in blocks to intervention or control using a balance algorithm based on practice list size, area deprivation, and clinical commissioning group [48]. When patients (45 y and older) consulted for OA during the study period and an OA Read code was recorded in their electronic GP records, a point-of-care electronic template was activated. This template provided a checklist of eligibility criteria and was used to prompt GPs to ask eligible patients the questions and to record the responses. Individual-level patient outcomes were measured by self-complete postal questionnaires administered to patients after their consultation and at 3, 6, and 12 mo follow-up (Fig 1) and by medical record review. The design was a professional-cluster intervention [49]: participating general practices provided informed consent, as “guardians” [50,51] for the patients in their care, that the practices were willing to enter the trial and to be randomised into either arm of the trial.

Ethical approval for this study was obtained from the Black Country Research Ethics Committee (reference number 11/WM/0093). There were no important changes to the study protocol after trial commencement.

### Participants

#### General practices

The study comprised Royal College of General Practitioners–approved “research ready” general practices that were using the EMIS consultation system at the time of recruitment and that were located in the West Midlands North region of England. Practices were approached and recruited over a 1-y period by the West Midlands North Primary Care Research Network (PCRN) to participate in the Primary Care Osteoarthritis Screening Trial (POST). Practices were provided with information on the general scope and purpose of the trial (practices were not given the exact nature of the screening in either arm of the trial prior to randomisation), the target population of interest, the anticipated 3- to 4-mo period of patient recruitment per practice, and the procedures that would need to be followed in the event of participating. Practices were provided with written and verbal information and had the opportunity to discuss participation with a GP (researcher facilitator or study principal investigator), research associate, and PCRN clinical studies officer.

#### Patients

Patients in the participating practices were prospectively identified for inclusion if they were aged 45 y or older, they consulted during the study period for clinical OA (defined using a pre-specified list of Read morbidity codes [code lists available from https://www.keele.ac.uk/mrr/]), their GP deemed them eligible and completed the electronic template during the consultation, and they provided full written informed consent to provide study data and to further contact at the time of post-consultation questionnaire completion. Consultations relating to a clinical diagnosis of OA (index consultation) could be first, new episode, or ongoing consultations. Patients with multiple OA consultations during the study period were sampled only once, at their first consultation in the study period. Patients were excluded by their GP at the point of template completion based on the following criteria: under active care for, or having a diagnosis of, depression and/or an anxiety disorder in the past 12 mo; vulnerable patient (on the Quality and Outcomes Framework mental health register or having a dementia diagnosis or terminal illness); nursing home resident; red flag pathology (recent trauma associated with significant injury; acute, red, hot swollen joint); or having inflammatory arthropathy, crystal disease, spondyloarthropathy, or polymyalgia rheumatica.

During the recruitment phase in each participating practice, GPs provided eligible patients in the consultation with a short information postcard introducing the study and notifying them that they would be contacted by the research team. Informatics staff from the PCRN performed weekly downloads of the names and addresses of eligible patients with template data (at least one response recorded) and mailed them a study pack (a letter from their general practice introducing the study, a patient information leaflet, and a self-completion questionnaire, including a consent form). The same information was provided to patients in both arms of the trial to reduce contamination bias [52]. Patients were asked for written informed consent for further contact and for their medical records to be accessed. A standard three-stage mailing approach was used (initial mailing, reminder postcard for non-respondents at 2 wk, and repeat study pack and reminder letter for non-respondents at 4 wk). Respondents consenting to further contact were sent follow-up questionnaires at 3, 6, and 12 mo. The same three-stage mailing procedure was used for follow-up questionnaires, with an additional short postal questionnaire at 6 wk comprising minimal data collection items for non-respondents (3, 6, and 12 mo), and telephone collection of the primary outcome at 8 wk (6 and 12 mo) for those who had not responded to a written questionnaire.

### Intervention

The intervention consisted of point-of-care anxiety and depression screening questions posed by the GP, prompted by the electronic template. The template for the intervention arm comprised five questions: a two-item ultra-brief depression tool (Patient Health Questionnaire [PHQ]–2; each item rated yes/no [32]) recommended by NICE for initial depression assessment in patients with a chronic physical health condition; a two-item ultra-brief anxiety assessment tool (Generalized Anxiety Disorder [GAD]–2 [53]), modified to use the same stem as the depression questions (“During the past month have you often been bothered by…”), with yes/no response options; and an item on current pain intensity rated on a 0–10 numerical rating scale (NRS) [54]. The GP recorded patients’ responses on the template. Negative responses to all ultra-brief depression and anxiety questions were used to rule out a potential depression or anxiety diagnosis. The template then signposted and encouraged GPs to follow NICE clinical guidelines on the management of OA, depression in adults with a chronic physical health problem [31], and anxiety [55]. At a post-randomisation meeting approximately 1 wk prior to the template being activated in the practice, a GP research facilitator (GPRF) employed by the PCRN explained and discussed the study procedures with GPs and practice staff. Brief face-to-face training was provided by the GPRF, explaining NICE-recommended evidence-based approaches to managing comorbid anxiety and depression, and hard copies of the screening questions and quick reference versions of the guidelines were placed in all consulting rooms in the intervention practices. The control condition was not disclosed to intervention practices. Reminder posters were placed in all consulting rooms to act as further prompts to the study.

The control arm received point-of-care pain intensity assessment by the GP, also prompted by the electronic template but containing only the item on current pain intensity. At the post-randomisation meeting with the GPRF, GPs were advised to follow their usual approach for responding to a patient’s pain intensity rating. No additional information or signposting on management was provided. The intervention condition was not disclosed to control practices.

In both arms, no additional treatment resources or services for depression, anxiety, or pain management were provided as part of this study. In pre-randomisation audits, the vast majority of practices in both arms reported having access to physiotherapy, rheumatology, pain clinic, orthopaedics, psychiatry, and cognitive behavioural therapy. Practices in both arms were contacted monthly by email and telephone, and received regular newsletters to encourage trial participation.

### Sample size

We aimed to detect a standardised effect size of 0.2 for the primary outcome of pain intensity time-averaged across all four follow-up time points (post-consultation and 3, 6, and 12 mo). Using ρ = 0.5 as an estimate of the autocorrelation of the primary outcome (current pain intensity) and specification of α = 0.05 and β = 0.10, the number required was 350 per arm (or 700 in total). This was adjusted for clustering effect between practices, taking into account unequal cluster sizes [56] using an anticipated intraclass correlation coefficient of 0.015 [57–59], average cluster size of approximately 30, and coefficient of variation around 0.5. Assuming 20% dropout, the inflation factor was 1.875, indicating that we would need 1,320 participants in the trial to detect an effect size of 0.2 with 90% power given the specified statistical parameters.

### Randomisation

General practices were randomly assigned to intervention or control on a 1:1 basis using a computer random number generator, with minimisation [48] used to constrict between-cluster variation in region (clinical commissioning group), area-level deprivation (Index of Multiple Deprivation [60]), and practice list size. General practices were randomised in six blocks of 5–10 practices as they agreed to take part. Final allocations of general practices were randomly selected by the independent statistician on the trial steering committee and passed to the PCRN, who installed the appropriate template into each practice and arranged for a GPRF to meet with each practice to introduce the screening template and study procedures. The chief investigator, principal investigator, trial statistician, and members of the administration team who inputted data from the study questionnaires were blinded to cluster allocation. Individual patients were not informed to which arm of the trial they were in.

### Outcomes

Outcome domains and validated measurement instruments were chosen to be consistent with recommendations for trials in OA [61] and chronic pain [62,63]. Individual patient outcomes were obtained from postal self-complete questionnaires and medical record review (consenting patients) covering the period up to 12 mo post-consultation.

The primary outcome for clinical effectiveness was patient-reported current pain intensity on a 0–10 NRS [54] across 12 mo post-consultation, i.e., analysis was undertaken across four time points: directly post-consultation and at 3, 6, and 12 mo. The question for this outcome was included in the point-of-care templates for both arms of the study. Secondary patient-reported outcomes measured across 12 mo post-consultation covered the following:

- pain experience: average (0–10 NRS), worst (0–10 NRS), and characteristic (0–100 NRS) pain intensity [54]; number of pain areas [64] and widespread pain (American College of Rheumatology definition [65] and Manchester definition [66]) as indicated on a blank manikin; patient assessment of change in pain (“Compared with when you first saw your doctor with this pain [X] months ago, how do you feel your pain is now?” [completely recovered, much better, better, same, worse, much worse])
- pain-related disability: interference with daily activities (0–10 NRS), recreational activities (0–10 NRS), and work (0–10 NRS); disability score (0–100) [54]; disability days from the Chronic Pain Grade (CPG) [54]; short-form Western Ontario and McMaster Universities Arthritis Index function subscale (for those with hip and/or knee only [0–32]) [67]
- mood: anxiety (GAD-7 [0–21]) [68]; depression (PHQ-8 [0–24]) [69,70]
- general health status: Medical Outcomes Study Short Form 12 Physical Component and Mental Component Scores (0–100) [71]

To evaluate treatment fidelity and acceptability, we asked patients in the post-consultation questionnaire to recall whether specific aspects were covered in their consultation (including discussion of mood and pain intensity), whether they perceived any irrelevant questioning, and whether they were satisfied with the consultation. The post-consultation questionnaire also collected descriptive information on demographic characteristics, socioeconomic status, living arrangement and availability of instrumental and emotional support [72], comorbidities, pain history, previous consultations (primary and secondary care), previous diagnoses of anxiety or depression, and pain catastrophizing [73].

To describe the patterns of care and identify any differences in these between the two arms, we collected and analysed healthcare use data using simple descriptive statistics. Information on healthcare use was collected by patient self-report (further GP consultations for the index pain complaint; other healthcare professional consultations for any reason [National Health Service (NHS) and private]; hospital visits for any reason [NHS and private]; and purchases of over-the-counter medicines, treatments, or appliances for any reason) and from information extracted from the primary care medical record (consultations for anxiety, depression, and OA; prescriptions for anxiety, depression, and OA medications; and referrals for counselling, psychology, psychiatry, physiotherapy, osteopathy, chiropractor, massage, orthopaedics, and pain clinic).

After completion of patient recruitment, GPs in each of the intervention and control practices were invited to complete a brief questionnaire that asked about the perceived ease of use of the template, its impact on length of consultation, doctor–patient communication, patient management, and excluding patients from the trial. All GPs consenting to further contact were invited to take part in audio-recorded interviews (group or individual; in person or telephone) with an experienced qualitative researcher (B. B.) to explore their questionnaire responses in greater detail. Full details of this will published separately.

### Trial analysis

Analysis of all numerical outcomes (including the primary outcome measure—current pain intensity) was by hierarchical linear mixed models with unstructured covariance, including general practice (at level 3) and individual participants (at level 2) as random effect variables (a logistic mixed model was used for categorical variables), with repeated measurements of assessment data per individual at level 1. A number of pre-specified covariates were included in the statistical models to help overcome potential selection and confounding bias. Fixed-effect covariates at level 3 included the three variables used in the minimisation procedure plus practice consultation rate (in the 12 mo prior to randomisation) for OA among patients aged 45+ y. Fixed-effect covariates at level 2 included age, sex, and time to respond following consultation (i.e., days between consultation date and mailing response date). In addition, for the primary analysis, the variable time (of follow-up assessment) was used as a level-1 fixed-effect covariate, as well as the interaction of time and level-2 and level-3 covariates. Analysis was performed on the basis of the intention-to-treat principle: evaluation was undertaken per cluster randomised allocation. Estimated mean responses between the two groups were compared (1) across all follow-up time points simultaneously as an aggregated summary (primary evaluation) and (2) across all time points distinctly (secondary evaluation being to determine whether effect differences were consistent or different at the three follow-up time points).

Sensitivity analyses of the primary outcome were performed on subsets of the study population. First, participants who provided consent to further contact and medical record review and had a recorded consultation pain template score were re-analysed including the electronic template pain score as an additional level-2 covariate. An extension of this model further included the following pre-specified additional baseline covariates: duration of complaint and body mass index (kg/m^2^). Second, we carried out a complier-average causal effect (CACE) analysis of the between-group differences in the primary outcome to estimate the effect of the intervention for participants whose GPs complied with the screening protocol; compliance was pre-specified as a “yes” response recorded in the template to either of the two depression or either of the two anxiety items or a “no” response across all four depression/anxiety items (all other combinations imply that the template was not sufficiently completed to aid in any diagnostic screening of anxiety/depression). Third, we carried out multiple imputation analysis using a more inclusive list of associated variables, including the same baseline pre-specified covariates but also all secondary outcome responses.

Pre-specified subgroup analyses focused on the interaction effect between study group and (1) age and (2) severity of pain (according to template completion). We hypothesized that, as found by Lin et al. [17], the effect of our screening intervention on reduction in pain would be less marked in those presenting with more severe pain. A copy of the statistical analysis plan is available on request.

### Patient and public involvement and engagement

Research users were involved in all stages of this trial, from grant application to final dissemination of results. Two users (J. B. and C. P.) were members of the trial steering committee, and a wider user group contributed to developing the trial design, study materials (including questionnaire and consent procedure), and intervention package.

## Results

### Participant flow

Participants were recruited from 4 July 2011 to 19 December 2012. A flow diagram illustrating the flow of practices and individual participants through the trial is given in Fig 1. In all, 45 general practices were randomised, with an overall mean list size of 6,694: 24 practices were randomised to the control group, and 21 practices to the intervention group (one practice in the intervention arm withdrew prior to patient recruitment). A total of 7,279 patients were identified as being potentially eligible (aged ≥45 y and receiving an OA Read code) for the trial by activation of the electronic template; 1,367 were deemed to be ineligible, the GP “escaped” (i.e., deactivated) the template for 2,198 patients, 1,582 were excluded for unknown reasons, and 90 eligible patients declined to take part, leaving 2,042 patients who had a completed template and were mailed a post-consultation questionnaire. The proportion of potentially eligible patients in whom the GP escaped the template was higher in the intervention group (*n =* 1,021, 33.6%) than in the control group (*n =* 1,177, 27.8%). In all, 1,412 (69.1%) participants responded to the post-consultation questionnaire: 911 (68.0%) in the control arm and 501 (71.3%) in the intervention arm. The mean time between the date of consultation and date of returning the post-consultation questionnaire was 24 d (interquartile range, 17–35 d; range, 9–149 d) in the control arm and 22 d (16–33; 3–106) in the intervention arm. Follow-up rates were similar in both arms, totalling 1,093 (77.4%) at 3 mo, 1,064 (75.4%) at 6 mo, and 1,017 (72.0%) at 12 mo; loss to follow-up was largely due to non-consent to further follow-up and non-response to mailing, though a small number of participants withdrew from the trial (reasons provided in the flow diagram).

### Practice and patient characteristics

Under the minimisation algorithm, more practices were allocated to the control group, and their total average practice list size was also higher than that of practices allocated to the intervention group (Table 1). Individual patients recruited from intervention and control practices had broadly similar characteristics (Table 2). In total, the average age of participants was 65 y, and 57% were female. The largest difference was in the proportion of patients reporting the pain episode to be their first: 40% in the control arm versus 33% in the intervention arm. For the subgroup of 1,035 study patients (644 [71%] in the control arm, 391 [78%] in the intervention arm) who consented to medical record review and had a consultation template pain score, the mean pain score was 6.33 (standard deviation, 2.04) in the intervention arm and 6.30 (2.10) in the control arm. In the intervention arm, 31.9% (125/392) of patients were recorded as having either anxiety or depressive symptoms, 20.2% (79/392) were recorded as saying “yes” to either of the two anxiety template questions, and 26.0% (102/392) were recorded as saying “yes” to either of the two depression template questions.

**Table 1:** Cluster-level baseline characteristics according to study group.

Characteristic	Control (*n* = 24)	Intervention (*n* = 20)
Clinical commissioning group*, *n* (percent)		
Region 1	6 (25.0%)	6 (30.0%)
Region 2	1 (4.2%)	2 (10.0%)
Region 3	5 (20.8%)	4 (20.0%)
Region 4	4 (16.7%)	4 (20.0%)
Region 5	1 (4.2%)	1 (5.0%)
Region 6	4 (16.7%)	1 (5.0%)
Region 7	3 (12.5%)	2 (10.0%)
Practice IMD score*, median (IQR)	16.8 (11.1–37.9)	20.3 (11.2–29.3)
Total practice list size*^†^**, mean (SD)**	7,397 (4,250)	5,850 (2,693)
**Practice list size for ages 45+ y**^‡^**, mean (SD)**	3,519 (2,170)	2,736 (1,244)
**Consultation rate for OA in past 12 mo (per 10,000 registered persons aged 45+ y), mean (SD)**	1,590 (959)	1,925 (746)

*Variable used in balance algorithm for randomisation.

^†^The sizes in the table relate to those used in the minimisation algorithm. Pre-audit figures are also available: the mean (SD) practice list size was 7,497 (4,420) for the control group, and 6,008 (2,664) for the intervention group.

^‡^Correlation between total practice size and practice size for persons aged 45+: 0.96.

IMD, Index of Multiple Deprivation; IQR, interquartile range; OA, osteoarthritis; SD, standard deviation.

**Table 2:** Participant baseline characteristics by study group.

Characteristic	Control (*n =* 911)	Intervention (*n =* 501)
**Age (years)**	65.4 (10.1)	65.6 (10.8)
**Female**	509 (55.9%)	290 (57.9%)
**White UK or European ethnicity**	888 (97.7%)	492 (98.2%)
**Living alone**	182 (20.0%)	108 (21.6%)
**Lack of emotional support**	41 (4.6%)	36 (7.2%)
**Lack of support with daily tasks**	57 (6.3%)	40 (8.0%)
**Currently in a paid job**	286 (31.4%)	166 (33.1%)
**Time off work in previous 6 mo**	102 (36.2%)	65 (39.4%)
**Self-reported body mass index (kg/m**^**2**^**)**	28.7 (5.6)	28.6 (5.1)
**Previous/current smoker**	457 (50.8%)	247 (49.3%)
**Daily/weekly alcohol consumption**	491 (54.4%)	245 (49.0%)
Area of pain at index consultation*		
Knee	496 (54.5%)	282 (56.3%)
Hip	224 (24.6%)	130 (26.0%)
Shoulder	155 (17.0%)	71 (14.2%)
Wrist or hand	121 (13.3%)	77 (15.4%)
Ankle or foot	126 (13.8%)	63 (12.6%)
Neck	56 (6.2%)	39 (7.8%)
Elbow	20 (2.2%)	20 (4.0%)
**First pain consultation episode**	355 (39.5%)	163 (32.8%)
**Duration of complaint**		
<3 mo	219 (24.7%)	118 (24.2%)
3–12 mo	261 (29.5%)	110 (22.5%)
1–5 y	269 (30.3%)	152 (31.2%)
>5 y	137 (15.4%)	108 (22.2%)
**Belief that pain started after accident/injury**	168 (18.6%)	89 (18.1%)
**Comorbidity**^†^	666 (73.1%)	370 (73.9%)
**Previous bone fracture**	375 (41.2%)	215 (42.9%)
**Previous falls in past 12 mo**	248 (27.4%)	147 (29.5%)
**Previous anxiety**	101 (11.2%)	40 (8.1%)
**Previous depression**	216 (23.9%)	102 (20.6%)
**Template pain score**^‡^	6.30 (2.10)	6.33 (2.04)

Data are given as mean (standard deviation) or *n* (percent).

*Does not add to 100% due to participants consulting for multi-joint pain.

^†^Any of the following self-reported conditions: previous heart attack or stroke, angina, raised blood pressure, diabetes, circulation problems in legs, cancer, liver disease, kidney disease, asthma/bronchitis, deafness, or eyesight problems.

^‡^Values based on 1,035 participants who consented to medical record review and who had baseline recorded template pain score (644 in the control group, 391 in the intervention group).

### Clinical effectiveness

The results for the analysis of the primary outcome measure (current pain intensity), including primary endpoint and secondary endpoint evaluations along with pre-specified ancillary analysis (sensitivity and subgroup analyses), are shown in Table 3. For the primary endpoint analysis there was a significantly higher average pain score over the four follow up time-points in the intervention group than the control group (mean difference 0.31: 95% CI 0.04, 0.59; effect size 0.15: 0.02, 0.29). The difference was not uniform across individual time-points; the largest difference of 0.52 was observed at 6 mo follow-up. All three sensitivity analyses showed similar results. The estimates for the subgroup analyses showed a statistically non-significant trend for decreasing difference in pain scores between intervention group and control group with increased age, but no statistical evidence of an interaction with pain severity recorded by the GP at the point of care. Secondary outcomes were consistent with the primary outcome measure in reflecting better outcomes as a whole for the control group than the intervention group (Table 4).

**Table 3:** Evaluation of the primary outcome measure (current pain intensity).

Analysis	Measure	Time point				Overall
		Post-consultation*	3 mo	6 mo	12 mo	
**Summary**	*n* control, *n* intervention	898, 493	701, 383	680, 374	644, 368	2,923, 1,618
	Control mean (SD)	5.3 (2.7)	4.5 (2.8)	4.2 (2.9)	4.0 (3.0)	4.6 (2.9)
	Intervention mean (SD)	5.7 (2.6)	5.0 (2.8)	4.8 (2.9)	4.3 (3.0)	5.0 (2.8)
**Main analysis primary endpoint**^†^	Mean difference	0.34	0.17	0.52	0.31	0.31^†^
	95% CI^‡^	0.02, 0.65	−0.19, 0.53	0.13, 0.90	−0.10, 0.72	0.04, 0.59
	*p*-Value	0.035	0.350	0.009	0.143	0.027
	Effect size	0.16	0.08	0.25	0.15	0.15
	95% CI^§^	0.01, 0.31	−0.09, 0.26	0.06, 0.43	−0.05, 0.35	0.02, 0.29
**Sensitivity analysis 1**	Mean difference	0.34	−0.02	0.45	0.20	0.25
	95% CI	0.01, 0.67	−0.41, 0.36	0.05, 0.84	−0.22, 0.62	−0.02, 0.52
	*p*-Value	0.044	0.903	0.027	0.353	0.071
**Sensitivity analysis 2**	Mean difference	0.41	0.07	0.36	0.25	0.37
	95% CI	0.04, 0.78	−0.36, 0.50	−0.12, 0.85	−0.24, 0.73	0.04, 0.69
	*p*-Value	0.029	0.753	0.137	0.324	0.030
**Sensitivity analysis 3**	Mean difference	0.39	0.19	0.57	0.39	0.36
	95% CI	0.05, 0.72	−0.20, 0.57	0.16, 0.98	−0.05, 0.83	0.06, 0.66
	*p*-Value	0.025	0.343	0.007	0.083	0.017
**Subgroup analysis 1**	Mean difference	−0.15	−0.35	−0.14	−0.32	−0.21
	95% CI	−0.44, 0.13	−0.67, −0.03	−0.47, 0.19	−0.70, 0.06	−0.46, 0.03
	*p*-Value	0.299	0.033	0.395	0.099	0.089
**Subgroup analysis 2**	Mean difference	0.03	0.08	0.04	0.06	0.05
	95% CI	−0.12, 0.19	−0.09, 0.24	−0.13, 0.21	−0.14, 0.26	−0.08, 0.18
	*p*-Value	0.673	0.365	0.655	0.569	0.468

Current pain intensity (0–10 numerical rating scale): 0 = no pain; 10 = pain as bad as could be. Values based on analysis of 4,541 patients with available data. Sensitivity analyses: (1) estimates based on a subgroup of the study population who consented to medical record review and had a baseline recorded template pain score (as well as available post-consultation data on duration of pain and body mass index); analysis based on a subset of 3,148 (1971 control, 1177 intervention) patients with available data by multilevel regression (as indicated in footnote ‡, with the addition of baseline pain template score, duration of pain, and body mass index as covariates); (2) “per protocol” evaluation through instrumental variable analysis using two-step least squares regression; (3) additional model adjustment for post-randomisation practice-level variables of number of patients mailed a post-consultation questionnaire and number of patients who returned a completed post-consultation questionnaire (to account for selection bias in recruitment uptake to the study). Subgroup analyses: multilevel linear regression analysis was carried out (as detailed in footnote ‡) including (1) group × age interaction term as the factor of interest—where age coefficients shown are based on units of 10 y (predicted mean difference in study population: 0.82 [age group 45–54 y], 0.55 [55–64 y], 0.41 [65–74 y], 0.10 [≥75 y])—and (2) group × pain (template pain score) interaction term as the factor of interest (predicted mean difference in study population: 0.17 [baseline pain template score 0–4], 0.37 [5–7], 0.61 [8–10]).

*Median (interquartile range) time between date of consultation and return of post-consultation questionnaire: control group, 24 d (17–35); intervention group, 22 d (16–33).

^†^Primary endpoint (overall pain intensity). The variance partition for the random coefficients was as follows: intraclass correlation coefficient = 0.011 (for between-practice variation) and intraclass correlation coefficient = 0.530 (for between-individual variation).

^‡^All analyses adjusted using general practice and repeated measures as random effects, and fixed-effect covariates at practice level (as outlined in Table 1) and patient level (age, sex, and time between consultation and post-consultation response). Estimates at the individual follow-up time points were obtained by inclusion of interaction terms for study group by time point of assessment. Evaluation was by multilevel linear regression analysis with longitudinal random slope parameterization.

^§^Estimated mean difference relative to SD of template pain score of 2.07.

SD, standard deviation.

**Table 4:** Secondary outcome measures.

Outcome	Measure	Time point				Overall
		Post-consultation	3 mo	6 mo	12 mo	
Average pain (0–10 NRS*) (*n =* 4,433^†^**)**	**Control**	6.4 (2.1)	5.7 (2.4)	5.0 (2.8)	4.7 (2.9)	5.5 (2.6)
	**Intervention**	6.7 (2.2)	6.1 (2.4)	5.6 (2.7)	5.1 (2.9)	5.9 (2.6)
	Mean diff. (95% CI)^‡^	0.25 (−0.01, 0.51)	0.20 (−0.13, 0.52)	0.56 (0.19, 0.93)	0.47 (0.06, 0.87)	0.29 (0.05, 0.52)
	*p*-Value	0.053	0.233	0.003	0.025	0.018
Worst pain (0–10 NRS*) (*n =* 4,454)	**Control**	7.7 (1.9)	7.0 (2.4)	6.0 (2.8)	5.6 (3.1)	6.7 (2.7)
	**Intervention**	7.8 (2.1)	7.2 (2.3)	6.6 (2.7)	5.8 (3.1)	7.0 (2.6)
	Mean diff. (95% CI)^‡^	−0.01 (−0.30, 0.28)	0.08 (−0.24, 0.40)	0.54 (0.19, 0.89)	0.52 (0.07, 0.97)	0.15 (−0.09, 0.40)
	*p*-Value	0.946	0.638	0.002	0.025	0.220
Interference with daily activities (0–10 NRS*) (*n =* 4,535)	**Control**	5.2 (2.8)	4.5 (3.0)	3.9 (3.1)	3.8 (3.1)	4.4 (3.0)
	**Intervention**	5.4 (2.9)	5.4 (3.0)	4.6 (3.0)	4.3 (3.2)	4.8 (3.0)
	Mean diff. (95% CI)^‡^	0.07 (−0.28, 0.41)	0.11 (−0.27, 0.50)	0.51 (0.11, 0.91)	0.39 (−0.04, 0.81)	0.20 (−0.11, 0.50)
	*p*-Value	0.706	0.570	0.013	0.074	0.203
Interference with recreational activities (0–10 NRS*) (*n =* 4,443)	**Control**	5.1 (3.1)	4.4 (3.2)	3.8 (3.2)	3.7 (3.2)	4.4 (3.2)
	**Intervention**	5.5 (3.1)	4.7 (3.3)	4.5 (3.2)	4.0 (3.3)	4.8 (3.3)
	Mean diff. (95% CI)^‡^	0.32 (−0.05, 0.70)	0.12 (−0.30, 0.54)	0.51 (0.09, 0.92)	0.38 (−0.06, 0.83)	0.34 (0.01, 0.67)
	*p*-Value	0.092	0.570	0.017	0.093	0.046
Interference with work (0–10 NRS*) (*n =* 4,440)	**Control**	4.6 (3.1)	4.1 (3.1)	3.7 (3.1)	3.5 (3.2)	4.0 (3.2)
	**Intervention**	5.1 (3.0)	4.4 (3.1)	4.3 (3.2)	3.9 (3.6)	4.5 (3.2)
	Mean diff. (95% CI)^‡^	0.32 (−0.05, 0.69)	0.09 (−0.30, 0.48)	0.50 (0.10, 0.91)	0.46 (0.00, 0.92)	0.32 (−0.01, 0.65)
	*p*-Value	0.092	0.655	0.015	0.049	0.059
WOMAC function subscale (0–32*) (*n =* 4,342)	**Control**	12.5 (7.6)	11.3 (7.8)	10.6 (7.6)	10.6 (7.9)	11.4 (7.8)
	**Intervention**	13.0 (7.7)	12.5 (7.6)	12.2 (7.9)	11.0 (8.1)	12.3 (7.9)
	Mean diff. (95% CI)^‡^	0.41 (−0.41, 1.23)	0.54 (−0.38, 1.46)	0.72 (−0.24, 1.69)	0.35 (−0.66, 1.36)	0.48 (−0.28, 1.24)
	*p*-Value	0.327	0.248	0.141	0.499	0.215
CPG pain subscale (0–100*) (*n =* 4,461)	**Control**	64.7 (19.4)	57.2 (22.9)	50.4 (26.6)	47.4 (28.1)	55.9 (25.0)
	**Intervention**	67.1 (19.5)	61.0 (22.8)	56.7 (25.5)	50.3 (28.4)	59.6 (24.6)
	Mean diff. (95% CI)^‡^	1.84 (−0.85, 4.53)	1.41 (−1.54, 4.36)	5.36 (2.16, 8.56)	4.59 (0.51, 8.66)	2.47 (0.05, 4.88)
	*p*-Value	0.181	0.348	0.001	0.027	0.045
CPG disability subscale (0–100*) (*n =* 4,457)	**Control**	49.8 (28.1)	43.6 (29.5)	38.0 (30.1)	36.4 (30.5)	42.7 (29.9)
	**Intervention**	53.2 (28.1)	46.7 (29.8)	44.7 (30.1)	40.0 (31.5)	46.8 (30.1)
	Mean diff. (95% CI)^‡^	2.30 (−1.05, 5.66)	1.11 (−2.68, 4.89)	5.13 (1.18, 9.07)	4.55 (0.32, 8.78)	2.87 (−0.22, 5.96)
	*p*-Value	0.179	0.567	0.011	0.035	0.069
**Disability days in past month (*n =* 4,432)**	**Control**					
	0–3	348 (39.0)	326 (46.7)	361 (55.1)	355 (57.4)	1,390 (48.6)
	4–7	139 (15.6)	111 (15.9)	91 (13.9)	86 (13.9)	427 (14.9)
	8–15	139 (15.6)	95 (13.6)	61 (9.3)	54 (8.7)	349 (12.2)
	16+	266 (29.8)	166 (23.8)	142 (21.7)	123 (19.9)	697 (24.4)
	**Intervention**					
	0–3	158 (32.5)	156 (40.8)	166 (46.2)	170 (49.7)	650 (41.4)
	4–7	88 (18.1)	69 (18.1)	65 (18.1)	54 (15.8)	276 (17.6)
	8–15	73 (15.0)	57 (14.9)	50 (13.9)	44 (12.9)	224 (14.3)
	16+	167 (34.4)	100 (26.2)	78 (21.7)	74 (21.6)	419 (26.7)
	OR (95% CI)^‡^	1.17 (0.68, 2.02)	1.15 (0.64, 2.06)	1.26 (0.67, 2.38)	1.42 (0.79, 2.55)	1.21 (0.77, 1.90)
	*p*-Value	0.569	0.650	0.474	0.248	0.406
**CPG**^§^ **(*n =* 4,411)**^‖^	**Control**					
	I	141 (15.9)	212 (30.4)	266 (41.0)	278 (45.2)	897 (31.5)
	II	280 (31.6)	175 (25.1)	133 (20.5)	118 (19.2)	706 (24.8)
	III	163 (18.4)	115 (16.5)	99 (15.3)	85 (13.8)	462 (16.2)
	IV	301 (34.0)	196 (28.1)	151 (23.3)	134 (21.8)	782 (27.5)
	**Intervention**					
	I	68 (14.1)	88 (23.1)	120 (33.4)	137 (40.2)	413 (26.4)
	II	117 (24.2)	104 (27.3)	72 (20.1)	68 (19.9)	361 (23.1)
	III	117 (24.2)	73 (19.2)	69 (19.2)	44 (12.9)	303 (19.4)
	IV	181 (37.5)	116 (30.5)	98 (27.3)	92 (27.0)	487 (31.1)
	OR (95% CI)^‡^	1.26 (0.84, 1.89)	1.22 (0.77, 1.93)	1.71 (1.00, 2.91)	2.12 (1.01, 4.43)	1.30 (0.89, 1.87)
	*p*-Value	0.265	0.407	0.048	0.047	0.176
**Perceived change (*n =* 2,977)**^‖^	**Control**					
	Completely recovered	—	26 (3.8)	43 (6.8)	63 (10.5)	132 (6.9)
	Much improved	—	98 (14.3)	135 (21.4)	141 (23.4)	374 (19.5)
	Improved	—	177 (25.8)	140 (22.2)	104 (17.3)	421 (22.0)
	No change	—	237 (34.6)	166 (26.4)	154 (25.6)	557 (29.0)
	Worse	—	121 (17.6)	112 (17.8)	115 (19.1)	348 (18.1)
	Much worse	—	27 (3.9)	34 (5.4)	25 (4.2)	86 (4.5)
	**Intervention**					
	Completely recovered	—	13 (3.5)	21 (6.0)	24 (7.2)	58 (5.5)
	Much better	—	52 (13.9)	47 (13.4)	69 (20.7)	168 (15.9)
	Somewhat better	—	70 (18.7)	62 (17.6)	57 (17.1)	189 (17.9)
	No change	—	139 (37.2)	109 (31.0)	101 (30.3)	349 (33.0)
	Worse	—	80 (21.4)	89 (25.3)	63 (18.9)	232 (21.9)
	Much worse	—	20 (5.4)	24 (6.8)	19 (5.7)	63 (6.0)
	OR (95% CI)^‡^	—	1.08 (0.71, 1.63)	2.17 (1.37, 3.42)	2.03 (1.11, 3.70)	1.46 (1.01, 2.12)
	*p*-Value	—	0.724	0.001	0.021	0.046
**Widespread pain (ACR definition) (*n =* 4,401)**	**Control**	221 (24.3)	149 (21.6)	136 (21.4)	150 (24.9)	656 (23.1)
	**Intervention**	128 (25.6)	91 (24.2)	94 (26.7)	82 (24.6)	395 (25.3)
	OR (95% CI)^‡^	0.95 (0.53, 1.70)	0.97 (0.50, 1.89)	1.28 (0.65, 2.50)	1.01 (0.51, 2.00)	1.03 (0.64, 1.64)
	*p*-Value	0.865	0.938	0.471	0.972	0.917
**Widespread pain (Manchester definition) (*n =* 4,401)**	**Control**	105 (11.6)	69 (10.0)	66 (10.4)	76 (12.6)	316 (11.1)
	**Intervention**	69 (13.8)	62 (16.5)	63 (17.9)	44 (13.2)	238 (15.2)
	OR (95% CI)^‡^	1.09 (0.51, 2.33)	2.77 (1.16, 6.61)	3.94 (1.61, 9.63)	1.24 (0.50, 3.11)	1.77 (0.97, 3.24)
	*p*-Value	0.816	0.022	0.003	0.641	0.063
GAD-7 (0–21*) (*n =* 4,359)	**Control**	5.1 (5.7)	4.6 (5.3)	4.6 (5.2)	4.6 (5.3)	4.8 (5.4)
	**Intervention**	5.6 (5.8)	5.2 (5.6)	5.7 (5.9)	5.5 (6.1)	5.5 (5.8)
	Mean diff. (95% CI)^‡^	0.18 (−0.49, 0.84)	0.34 (−0.36, 1.05)	0.44 (−0.28, 1.16)	0.61 (−0.14, 1.37)	0.34 (−0.27, 0.94)
	*p*-Value	0.603	0.341	0.230	0.112	0.272
PHQ-8 (0–24*) (*n =* 4,376)	**Control**	6.0 (6.0)	5.2 (5.7)	5.3 (5.7)	5.4 (6.0)	5.5 (5.8)
	**Intervention**	6.4 (6.1)	6.0 (6.1)	6.6 (6.3)	6.0 (6.1)	6.3 (6.1)
	Mean diff. (95% CI)^‡^	0.30 (−0.40, 1.00)	0.52 (−0.22, 1.26)	0.74 (−0.02, 1.49)	0.36 (−0.41, 1.14)	0.45 (−0.20, 1.09)
	*p*-Value	0.402	0.172	0.055	0.360	0.176
**SF-PCS (0–100**^¶^**) (*n =* 4,263)**	**Control**	36.0 (11.1)	37.9 (11.4)	39.3 (11.8)	39.1 (11.9)	37.9 (11.6)
	**Intervention**	35.5 (10.5)	36.3 (10.8)	36.3 (11.3)	38.1 (11.6)	36.4 (11.0)
	Mean diff. (95% CI)^‡^	0.24 (−1.07, 1.55)	−0.23 (−1.63, 1.17)	−1.77 (−3.22, −0.32)	−0.66 (−2.25, 0.93)	−0.33 (−1.54, 0.89)
	*p*-Value	0.717	0.749	0.017	0.419	0.598
**SF-MCS (0–100**^¶^**) (*n =* 4,263)**	**Control**	49.9 (11.4)	49.6 (11.5)	49.0 (11.7)	49.2 (11.3)	49.5 (11.5)
	**Intervention**	49.1 (11.2)	48.4 (11.5)	47.6 (12.0)	48.8 (11.6)	48.5 (11.6)
	Mean diff. (95% CI)^‡^	−0.61 (−1.98, 0.76)	−0.79 (−2.26, 0.69)	−0.12 (−1.62, 1.39)	−0.32 (−1.88, 1.25)	−0.50 (−1.73, 0.72)
	*p*-Value	0.383	0.295	0.878	0.691	0.418

Data are mean (standard deviation) unless otherwise indicated.

*Higher scores represent a worse rating.

^†^Total number of available longitudinal data that were analysed (in each case).

^‡^All analyses adjusted using general practice and repeated measures as cluster-level random effects, and fixed-effect covariates at practice level (as outlined in Table 1) and patient level (age, sex, and time between consultation and post-consultation response). Analysis was carried out by linear/logistic mixed models.

^§^CPG categories: I—low disability, low intensity; II—low disability, high intensity; III—high disability, moderately limiting; IV—high disability, severely limiting.

^‖^Analysis was carried out via ordinal mixed model.

^¶^Lower scores represent a worse rating.

ACR, American College of Rheumatology; CPG, Chronic Pain Grade, diff., difference; GAD, Generalized Anxiety Disorder; NRS, numerical rating scale; OR, odds ratio; PHQ, Patient Health Questionnaire; SD, standard deviation; SF-MCS, Medical Outcomes Study Short Form 12 Mental Component Score; SF-PCS, Medical Outcomes Study Short Form 12 Physical Component Score; WOMAC, Western Ontario and McMaster Universities Arthritis Index.

The proportion of patients reporting that the GP asked irrelevant questions in the consultation was low and similar in both arms (41 [8.3%] in intervention group, 50 [5.6%] in control group) (Table 5). The proportion of patients not satisfied with the consultation was higher in the intervention group than in the control group (71 [14.5%] and 89 [9.9%], respectively).

**Table 5:** Acceptability and fidelity of screening from participants’ post-consultation questionnaires.

Item	Control (*n =* 911)	Intervention (*n =* 501)	*p*-Value
**Topics doctor asked about**			
How long you have had your pain	696 (76.4%)	368 (73.5%)	0.219*
The intensity of your pain	674 (74.0%)	348 (69.5%)	0.069
How your pain interferes with daily activities	410 (45.0%)	236 (47.1%)	0.448
Your mood	90 (9.9%)	157 (31.3%)	<0.001
**Doctor asked irrelevant questions**			
No	743 (82.6%)	391 (79.1%)	
Unsure	106 (11.8%)	62 (12.6%)	
Yes	50 (5.6%)	41 (8.3%)	0.117
Irrelevant questions on mood^†^	3	7	
**Satisfied with consultation**			
Yes	676 (75.5%)	342 (69.8%)	
Unsure	130 (14.5%)	77 (15.7%)	
No	89 (9.9%)	71 (14.5%)	0.025

Data are given as *n* (percent).

*χ^2^ test.

^†^From free-text responses to an open-ended question on irrelevant questions, completed by 29 and 27 patients in the control and intervention groups, respectively.

### Patterns of healthcare use

GP visits for depression, anxiety, and OA were higher among intervention patients, as were visits to other NHS professionals for any reason. However, there were no other significant between-group differences in NHS or private medical resource use (S1 Table).

## Discussion

This pragmatic cluster randomised trial in UK primary care provides no evidence for a beneficial effect on patient-reported outcomes of implementing active screening for anxiety and depression in patients consulting with OA. Participants in the intervention group reported significantly higher average pain scores over the four follow-up time points than participants in the control group, with key secondary outcomes also reflecting better outcomes in the control group.

Despite national guidelines advocating screening for anxiety and depression in high-risk groups [30], including those with OA and painful conditions, this is the first large pragmatic primary-care-based trial to our knowledge to investigate the utility of this approach. Our findings cast doubt on the validity of national guidance about the usefulness of routine screening for anxiety and depression in people with long-term conditions in primary care. In the absence of direct clinical trial evidence, the introduction in 2006 of financial incentives for annual depression screening in people with coronary heart disease and diabetes in the UK has provided a natural experiment, albeit without random allocation of controls, on the effects of implementing screening in routine care for defined high-risk groups. Modest increases in the rate of new depression diagnoses and in antidepressant prescriptions were observed [74,75], although the impact of these changes and their relation to patient outcomes remain unclear. Screening for depression in patients with diabetes and heart disease has subsequently been withdrawn from the Quality and Outcomes Framework component of the general practice contract.

### Strengths and limitations

This study has a number of strengths and adds to the growing literature in this area. This large primary-care-based trial helps address the evidence gap using validated screening tools with established diagnostic accuracy in a high-risk population without a current depression diagnosis. The point-of-care screening was conducted by the patients’ GP during an unsolicited consultation for OA. Methodologically, we had minimal cluster attrition, with only one practice withdrawing from the trial, and had high rates of follow-up from participants.

A number of study limitations need to be noted. This trial experienced an imbalance in allocation to clusters, with the intervention arm being allocated fewer practices that were smaller in size (average practice size for control practices = 7,397, average size for intervention practices = 5,850). However, this did not impact the statistical power of the study and is unlikely to have introduced significant bias. Selection bias is always an important consideration in cluster trials, and the trial was designed to minimise this [49]. Although this study recruited large numbers of patients to both arms, the proportion of potentially eligible patients who were screened by the GP and mailed a post-consultation questionnaire was higher in the control group (*n =* 1,339, 31.6%) than in the intervention group (*n =* 703, 23.1%). The differential recruitment between arms was unrelated to patients’ age, sex, or whether their joint problem was given a diagnosis of OA (Read code N05) or a symptom code related to a clinical diagnosis of OA (a proxy for the severity and prognosis of the joint problem) (Table 6). Mean pain score recorded at the time of consultation was similar in both arms. However, we cannot know whether the differences between arms in the distribution of prognostic factors (e.g., longer duration of episode, more previous episodes, more widespread pain, greater interference with daily activities, more severe anxiety and depression symptoms [76,77]) measured for the first time at post-consultation questionnaire were also present at the time of the consultation.

**Table 6:** Recruitment rates by arm: overall, and by age, sex, and diagnostic code within arm.

Group	Potentially eligible patients for whom the GP escaped the template*		Potentially eligible patients mailed a post-consultation questionnaire^†^		Response among patients mailed post-consultation questionnaire^‡^	
	Control	Intervention	Control	Intervention	Control	Intervention
**Overall**	27.8% (1,177/4,238)	33.6% (1,021/3,041)	31.6% (1,339/4,238)	23.1% (703/3,041)	68.0% (911/1,339)	71.3% (501/703)
**By age**						
45–64 y	28.3% (605/2,135)	34.8% (535/1,538)	31.7% (676/2,135)	22.1% (340/1,538)	61.7% (417/676)	65.3% (222/340)
65–84 y	25.6% (481/1,882)	31.7% (436/1,375)	33.4% (628/1,882)	24.9% (343/1,375)	75.6% (475/628)	76.7% (263/343)
85+ y	41.2% (91/221)	39.1% (50/128)	15.8% (35/221)	15.6% (20/128)	54.3% (19/35)	80.0% (16/20)
**By sex**						
Male	29.0% (518/1,788)	33.7% (437/1,295)	31.8% (569/1,788)	24.2% (313/1,295)	70.7% (402/569)	67.4% (211/313)
Female	26.9% (659/2,450)	33.4% (584/1,746)	31.4% (770/2,450)	22.3% (390/1,746)	66.1% (509/770)	74.4% (290/390)
**By diagnostic code**						
N05	18.5% (165/893)	27.8% (193/694)	43.3% (387/893)	36.5% (253/694)	xx/387	xx/253
Not N05	30.3% (1,012/3,345)	35.3% (828/2,347)	28.5% (952/3,345)	19.2% (450/2,347)	xx/952	xx/450

Data are given as percent (*n/N*) (just *n/N* where data were not available).

*Escaped population/potentially eligible population.

^†^Mailed population/potentially eligible population.

^‡^Post-consultation questionnaire respondents/mailed population.

GP, general practitioner; xx, data not available.

Whilst a degree of selection bias may have occurred, we do not believe it capable of overturning the key finding of this study, namely, a lack of demonstrable benefit from implementing screening for anxiety and depression for patients with OA consulting their GP.

### Results in relation to other studies

To date, the evidence supporting screening for anxiety and depression in patients with OA is limited, although evidence from a previous single clinical trial of a collaborative care approach suggests that successfully managing comorbid major depressive disorder in patients with OA can improve pain, function, and quality of life [16–18]. Some caution is needed when directly comparing existing evidence with the current study, which took a different overall approach. In our study, patients consulting with OA were screened to identify depression or anxiety with the intention of providing treatment (if deemed necessary), whilst the study by Lin et al. successfully used a collaborative care approach (an intensive intervention, with case management and active follow-up) in those already identified and diagnosed with depression. As our study failed to improve depression and anxiety outcomes, it would be unlikely to have an impact on pain. Whilst more research has been conducted among those with long-term conditions other than OA, the recommendation of routine depression screening in general [28] and for case-finding in high-risk patients with diabetes [42], cancer [43], and coronary heart disease [44] has been criticised as premature. As such it is not currently clear that screening, even if conducted alongside collaborative care management of treatment, would be effective.

The findings from the Primary Care Osteoarthritis Screening Trial are generally consistent with the limited effects reported by other studies using point-of-care prompts to influence clinical behaviour [78,79]. A recent study investigating the impact of a pop-up electronic template to collect additional data during GP consultations found considerable variation between individual clinicians and for different quality indicators of OA care [41]. One explanation for these findings is that screening may occur in isolation and not fit naturally into the GP consultation. Furthermore, one study [80] suggested that, to be successful, screening needs to operate within structured pathways that can be accommodated within available systems and resources. Other evidence suggests that patients may prefer to separate physical and mental health problems within the context of long-term condition management, finding a preference among patients for discussing emotional problems in a separate therapeutic space [81].

### Implications for clinicians, policy makers, and future research

Physician behaviour can be expected to influence the expression of emotional cues and concerns by patients, and there is evidence that both elicitation and recognition are highly variable between practitioners [82]. Closed questions on psychosocial issues may facilitate the expression of emotional cues and concerns [83,84]. In this alternative perspective of screening, in which the purpose is to facilitate a more holistic assessment in order to improve pain and functional outcomes, the beneficial effects of raising the issue of feelings of anxiety and depression within the OA consultation need not be restricted to the relatively small minority of patients ultimately diagnosed with anxiety or depressive disorder who access and receive high-quality mental healthcare (the screen–diagnose–treat pathway). Gask and Coventry [85] argue that whilst practitioners can be trained to take a more holistic approach, we need to acknowledge the degree of complexity present in the healthcare system that works against achieving satisfactory implementation and outcomes from person-centred mental healthcare.

The results of this study demonstrate that current guidance recommending depression screening of high-risk individuals needs to be questioned, especially given the negative outcome in patients with OA. Such outcomes may also apply to other long-term conditions, although caution is needed before extending our findings to other pain-related conditions, such as fibromyalgia, where the relation between anxiety and depression and the clinical syndrome may be different to that seen in OA.

## Conclusion

In this study, we observed that encouraging GPs to routinely ask screening questions for anxiety and depression of patients consulting for clinical OA (and then to follow guideline-recommended care for OA and mental health) had no benefit on patient-reported pain and functional outcomes over 12 mo.

## Supporting information

S1 Data Access to services reported by practices in the pre-audit. (DOCX)

S1 Table Healthcare-related resource use over 12 mo, by group. (DOCX)

S1 Text Study protocol. (DOCX)

S2 Text CONSORT checklist. (DOC)

## Abbreviations

GAD: Generalized Anxiety Disorder
GP: general practitioner
GPRF: general practitioner research facilitator
NHS: National Health Service
NICE: National Institute for Health and Care Excellence
NRS: numerical rating scale
OA: osteoarthritis
PCRN: Primary Care Research Network
PHQ: Patient Health Questionnaire
