# Supplementary material for: The effects of implementing a point-of-care electronic template to prompt routine anxiety and depression screening in patients consulting for osteoarthritis (the Primary Care Osteoarthritis Trial): A cluster randomised trial in primary care
Source: PLoS Med. 2017 Apr 11;14(4):e1002273. doi: 10.1371/journal.pmed.1002273 (PMC5388468; doi:10.1371/journal.pmed.1002273)
Supplement: S1 Data — (DOCX) [file pmed.1002273.s001.docx]

**S1 Data:** Access to services reported by practices in the pre-audit

|  | Intervention (n=20) | Control (n=22) |
| --- | --- | --- |
| Physiotherapy | 20 *(100)* | 22 *(100)* |
| Rheumatology | 20 *(100)* | 22 *(100)* |
| Pain clinic | 20 *(100)* | 21 *(95)* |
| Orthopaedics | 19 *(95)* | 22 *(100)* |
| Psychiatry | 17 *(85)* | 19 *(86)* |
| CBT | 17 *(85)* | 17 *(77)* |
| Psychiatric nurse | 16 *(80)* | 12 *(55)* |
| Lay counsellor | 7 *(35)* | 5 *(23)* |
| Computerised CBT | 6 *(30)* | 4 *(18)* |
| Telephone counselling | 4 *(20)* | 5 *(23)* |
| Peer support programme | 2 *(10)* | 3 *(14)* |
