# Supplementary material for: The effects of implementing a point-of-care electronic template to prompt routine anxiety and depression screening in patients consulting for osteoarthritis (the Primary Care Osteoarthritis Trial): A cluster randomised trial in primary care
Source: PLoS Med. 2017 Apr 11;14(4):e1002273. doi: 10.1371/journal.pmed.1002273 (PMC5388468; doi:10.1371/journal.pmed.1002273)
Supplement: S1 Table — (DOCX) [file pmed.1002273.s002.docx]

**S1 Table:** Healthcare-related resource use over 12 months, by group

|  | **Mean (SD) number of consultations** | | | | | |
| --- | --- | --- | --- | --- | --- | --- |
|  | **n** | **Control** | **n** | **Intervention** | **Mean diff (95% CI)** |  |
| *Healthcare professional visits* | | | | | | |
| *Primary care*^†^: |  |  |  |  |  |  |
| General practitioner | 753 | 2.42 (2.07) | 424 | 2.86 (2.25) | 0.431 (0.178,0.714) |  |
| Practice nurse | 753 | 0.03 (0.18) | 424 | 0.03 (0.17) | 0.004 (-0.015,0.026) |  |
| Other | 753 | 0.07 (0.34) | 424 | 0.04 (0.20) | -0.027 (-0.057,0.004) |  |
| *Secondary care*^††^*:* |  |  |  |  |  |  |
| Appointments NHS consultant | 633 | 2.07 (4.38) | 352 | 1.69 (2.94) | -0.377 (-0.813, 0.109) |  |
| Appointments with private consultant | 633 | 0.44 (1.93) | 352 | 0.41 (1.70) | -0.027 (-0.243, 0.241) |  |
| Appointments with ‘other’ professionals in NHS hospitals | 633 | 0.13 (0.53) | 352 | 0.26 (1.24) | 0.129 (0.022,0.284) |  |
| Appointments with ‘other’ professionals in private hospitals | 633 | 0.06 (0.56) | 352 | 0.05 (0.50) | -0.006 (-0.067,0.069) |  |
| Inpatient stay(days) | 633 | 1.64 (3.19) | 352 | 1.65 (2.91) | 0.003 (-0.377, 0.389) |  |
| *Prescriptions*^†a^ |  | n (%) |  | n (%) |  |  |
| Proportion received anxiety/depression | 796 | 150 (18.9%) | 439 | 100 (22.8%) | ( - ) |  |
| Proportion received OA prescriptions | 796 | 567 (71%) | 439 | 343 (78%) | ( - ) |  |
|  |  | n (%) |  | n (%) |  |  |
| Over-the-counter medicines/treatments ^a††^ | 633 | 373 (58.9) | 352 | 201 (57.10) | ( - ) |  |
| Investigations and treatments ^a††^ | 633 | 372 (58.8) | 352 | 197 (55.9) | ( - ) |  |
| † Data based on medical records review of available data and includes anxiety/depression and OA related healthcare use  †† Data based on self-reported questionnaires at 6 and 12 months and includes healthcare use for any health reason  a The number (%) of participants reporting usage within the procedures, investigations, procedures, over-the-counter medicines and prescribed medication categories are reported instead of mean (SD) because of multiple usage, purchases and/or prescriptions over 12 months. | | | | | | |
