# Supplementary material for: The effects of implementing a point-of-care electronic template to prompt routine anxiety and depression screening in patients consulting for osteoarthritis (the Primary Care Osteoarthritis Trial): A cluster randomised trial in primary care
Source: PLoS Med. 2017 Apr 11;14(4):e1002273. doi: 10.1371/journal.pmed.1002273 (PMC5388468; doi:10.1371/journal.pmed.1002273)
Supplement: S1 Text — (DOCX) [file pmed.1002273.s003.docx]

# Study Protocol

**Primary care Osteoarthritis Screening Trial (POST)**

**ISRCTN: (apply for after ethical approval)**

# Author list (for purposes of protocol paper)

Nicholl BI, Mallen CD, Belcher J, Packham J, Jowett S, Whitehurst D, Whitehurst T, Thomas I, Clements C, Bratt A, Hay E, Peat GM.

**Research Team**

| **Member** | **Role** |
| --- | --- |
| Christian Mallen | Principal Investigator |
| George Peat | Chief Investigator |
| Elaine Hay | PI of NIHR Osteoarthritis Programme Grant |
| Barbara Nicholl | Research Associate and Study Coordinator |
| John Belcher | Statistician |
| Sue Jowett | Health Economist |
| Jon Packham | Senior Lecturer in Rheumatology |
| Tracy Whitehurst | Informatics Manager |
| Ian Thomas | Informatics Systems Developer |
| Samantha Hunt | Clinical Studies Officer |
| Charlotte Clements | Clinical Research Support Coordinator |
| Alicia Bratt | Administrative Assistant |

# Background

Osteoarthritis (OA) is a chronic condition affecting the joints of older people. It is often associated with functional limitation and persistent pain, both of which can impact on mood and quality of life. OA is one of the most common reasons for primary care consultation in the UK, with approximately 1 million seeking treatment each year [1]. Despite it being a prevalent disorder the ability to effectively treat and maintain an improvement in OA symptoms in primary care is limited [2]. One reason for this may be that the majority of prognostic indicators for the course of OA are not modifiable (e.g. age, gender and symptom duration). One potentially modifiable factor associated with the outcome of OA is depressive symptoms [3]. In individuals with persistent pain conditions, such as OA, depression has been shown to occur up to four times more frequently than in individuals without persistent pain [4-6]. Furthermore, the results of a randomised controlled trial showed that treating major depression in patients with OA resulted in improvements in OA outcomes, including pain and function, and quality of life [7,8]. However, the detection of depressive symptoms in primary care is poor, particularly in those patients with a chronic physical illness [9,10]. Depressive symptoms that do not reach criteria for major depression, often termed “sub-threshold” or “mild” depression, are still considered to be distressing and disabling, particularly for patients with a chronic disease such as OA, and stepped care of “active monitoring” or treatment is recommended [11].

Screening for depression as a routine assessment endeavours to improve identification of depressive symptoms, and consequently facilitate implementation of the management of depressive symptoms. Screening for depressive symptoms during primary care consultations for diabetes and coronary heart disease patients, using two brief questions [12], has been stipulated as part of the Quality Outcomes Framework for UK primary care practice since 2006. Indeed, screening and subsequent treatment of depressive symptoms in patients with diabetes has been shown to be associated with healthcare cost savings [13], and it is likely that a similar finding would be observed in this trial, amongst patients with OA. Recent literature reviews have recommended that such screening is also carried out in high risk groups with comorbid medical conditions [14,15], including chronic painful conditions [9,16]. The American College of Preventative Medicine goes as far as to state that primary care providers should screen all adults for depression [17], yet in order for screening to be beneficial, evidence suggests that effective systems for the diagnosis, treatment and management of depression should be in place [18,19]. Such recommendations for depressive symptom screening have now been included in the National Institute for Clinical Excellence (NICE) guidelines for holistic OA management [20]; however, the extent of their implementation is yet to be ascertained. NICE guidelines for depression in adults with a chronic physical health problem [11] give details of how such comorbid depressive symptoms should be identified, treated and managed [21]. Ultra-short screening tools (two to three items) have been shown to be useful at identifying up to eight out of 10 probable cases of depression with a high false positive rate noted [22]. However, screening is accepted as a method of identifying depressive symptoms that warrant a subsequent more detailed assessment to determine their severity [11,22]. A recent study that used the 2-item depression screen in patients with musculoskeletal pain supported these findings [23], highlighting the importance of detecting depressive symptoms in chronic pain patients, even those that are considered to be sub-threshold or mild symptoms, rather than just screening for diagnosable depressive disorders. This observational study of 428 patients aged 50 years and older consulting with non-inflammatory musculoskeletal pain found that 18.2% (n=78) screened positive for depression using the PHQ-2 [23]. It is unlikely that screening by itself will result in improved symptoms of either depression or pain, rather it is anticipated that identification of depressive symptoms would result in altering care to address and, where appropriate, initiate treatment, for such symptoms [24]. It is this screening combined with collaborative care of comorbid depressive symptoms that appears to be successful in reducing OA related symptoms [7,8]. However, screening continues to be recommended in clinical guidelines [20], yet how beneficial such a screening and subsequent modification of care approach is for OA patients with sub-threshold or mild depressive symptoms has yet to be determined.

The majority of work concerning comorbid mood disorders in primary care, has investigated depression, however, anxiety has been shown to be common in primary care patients (14.6% to 19.5%) [25,26]. Similarly, evidence suggests that anxiety might be more common than depression amongst patients with OA (e.g. 46.4% vs. 29.2% [27]; 48.9% vs. 26.5% [28]; 44.7% vs. 34.5% [29]. However, less than half of anxious cases are estimated to be recognised by their GP [27]. It is common for anxiety to lead to the development of depression [30] or co-occur with depressive symptoms [31] in primary care patients; and to have a potentially negative impact on well-being [32]. Consequently, screening for anxiety symptoms in OA primary care patients has been recommended as the first step in improving patient outcomes [20] and to help prevent the development of depressive disorders [33,34]. The recent development of a 2-item screening tool for generalised anxiety disorder (GAD-2) [26] also efficiently screens for three other common anxiety disorders (panic disorder, social anxiety disorder, and posttraumatic stress disorder) and is suitable to detect anxiety symptoms in patients across a continuum of anxiety symptoms.

We shall undertake a cluster randomised trial that prompts the recommended holistic assessment of OA [20] by screening for both anxiety and depression in an OA primary care population. This will allow us to determine the effectiveness of screening in terms of an improvement in pain related outcomes and additionally allow us to determine the cost effectiveness of such a screening intervention, as recommended in a recent systematic review of literature regarding screening for depression in primary care [35]. There is growing evidence of the futility of screening for depressive symptoms and disorders in the primary care setting without an intensive collaborative care treatment plan in place [36,37]. Nevertheless, screening for “case finding” is recommended in the GP contract for patients with chronic heart disease and diabetes [38]; it is also recommended in NICE guidelines for patients with a chronic physical health problem [11]. However, it is important to emphasise that this trial is not intended to instigate a system of case-finding and management of major depressive or anxiety disorder requiring a highly complex intervention incorporating the provision of best-evidence treatment for these disorders; rather we are interested in the effect on OA care and outcomes of introducing structured prompts to alert GPs to the possible presence of concurrent depressive and anxiety symptoms as part of a holistic assessment in patients presenting to their GP with clinical OA or peripheral joint pain.

# Aims

The overall aim of this trial is to develop and test a systems intervention to prompt holistic OA treatment in primary care; first and foremost with the recognition of concurrent depressive and anxiety symptoms. This will allow us to investigate whether routinely incorporating brief screening questions for depressive and anxiety symptoms in OA patients results in an improvement in their OA care and outcomes. The clinical and cost-effectiveness of such an intervention compared to a control arm of usual care will be investigated.

## Primary objective

The primary objective of the trial is to determine whether screening for generalised anxiety and depressive symptoms in patients who consult their GP with OA or peripheral joint pain results in an improvement in their pain-related outcomes. Specifically, we hypothesise that patients who are screened for depressive and anxiety symptoms in their GP consultation will have an improvement in their self-reported current pain intensity and pain interference with daily activity ratings (numerical rating scale 0-10) over the 12 months following their consultation, compared to patients in the control arm. Growth curve models will be used to summarise the pattern of response over time between the groups. Changes in slope and intercept will reflect differences in rate of change and mean scores over the 12 months.

## Secondary objective

A secondary objective of the trial will be to evaluate the cost utility of screening for depressive and anxiety symptoms in patients with OA.

# Methods/Design

This study is designed as a cluster randomised controlled trial with two arms. Both arms will ask a standard set of questions during the consultation; however, the content of the template will vary between the two trial arms. The template in the intervention arm will include a total of five questions (two each for depression and anxiety screening, and a fifth question on pain intensity), the template for the control arm will include only the one question on pain intensity; further details of the screening template are provided below. It is termed as a “systems” intervention to incorporate both the screening template and subsequent approach to treatment that the GP follows. The treatment itself will not be implemented as part of the trial protocol; instead the recommended treatment course will be reinforced with GPs in the intervention arm prior to the study commencing (further details provided below). A summary of the study design is illustrated in Figure 1.

A cluster randomised trial rather than an individual randomised trial was chosen for both scientific and practical reasons. GPs are likely to find it difficult to act differently towards patients if they were individually randomised to control and intervention arms during the consultation and therefore contamination between the two arms would be likely. This trial can be thought of as a professional-cluster intervention type [39], in that the intervention involves professional activity during consultation and although the patient can opt out of their data being used, the intervention is still likely to have an effect on them since it involves introducing specific questions to an OA/peripheral joint pain consultation. To this extent the intervention or control template has to be introduced at a practice level since it involves using and comparing the outcome from practice level services.

A period of “run-in” will be adopted to ensure that all study procedures run smoothly and according to protocol. Further details of this period are given below.

**Figure 1:** Flowchart of study design


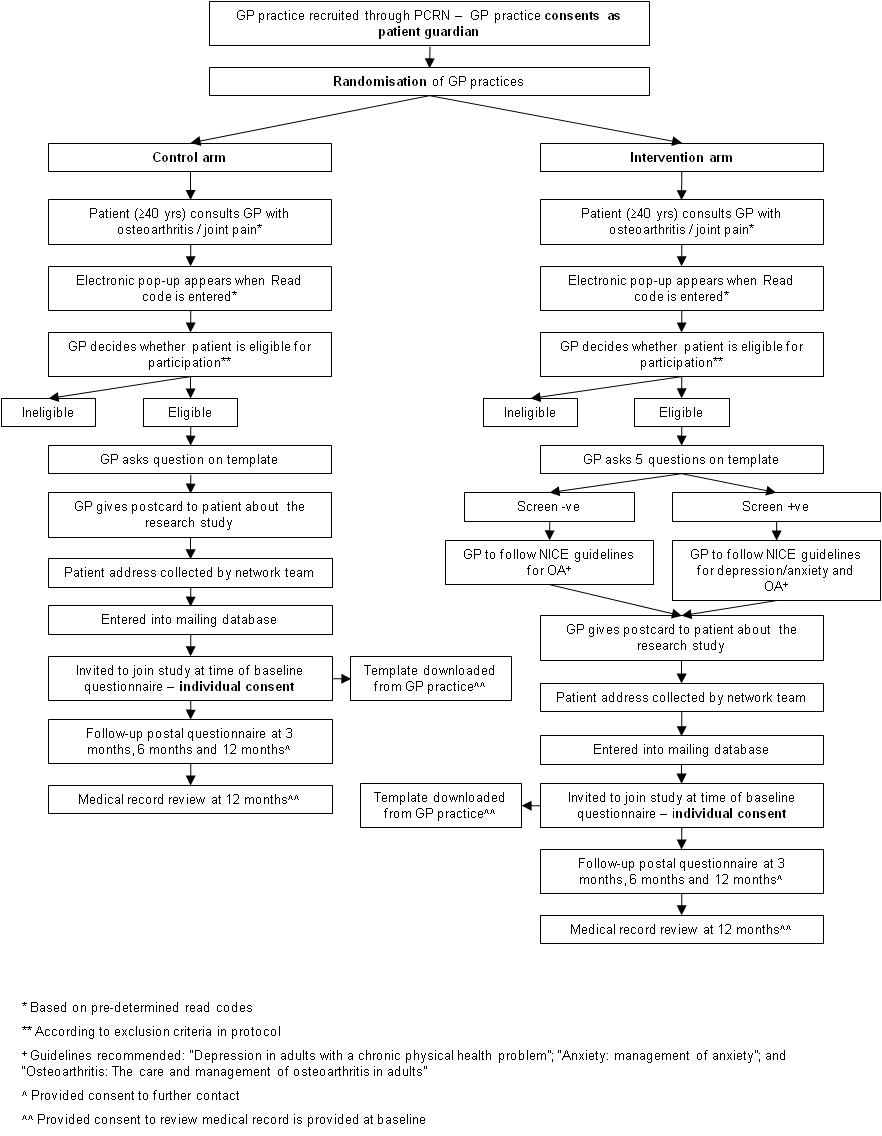


***Minimising systematic bias***

The threat of selection and recruitment bias in cluster randomised trials is high [40]. A number of steps have been taken in the design of this trial to minimise these threats:

- All GP practices will be given the same information about the trial prior to randomisation. This will be brief information about the study and will not include specific details of the study hypotheses. By not raising the profile of depressive and anxiety symptom screening prior to randomisation we anticipate that selection bias of GP practices agreeing to take part will be limited as will contamination about anxiety/depressive screening to GPs in practices that are subsequently randomised to the control arm.
- The electronic template will only be active in each GP practice for approximately three months and will apply to all GPs working in that practice. Therefore any extra work that may be considered as a result of the screening template will not fall on only one GP and the relatively short time period will mean that GPs are less likely to preferentially select patient’s based on their work demands.
- The study name was purposely chosen as it does not include any psychological terms; therefore eluding direct information about the study hypotheses, both to GPs and to patients.
- The electronic screening template is designed to automatically “pop-up” when a Read code for OA/peripheral joint pain (from a pre-determined list) is entered onto an eligible patient’s electronic record. This process has worked well in a previous study carried out at this research centre [23]. This method omits any human error on the GP’s part to forget to ask an eligible patient the screening questions.
- If a GP deems a patient as ineligible to take part they will be asked to give a reason for this exclusion before exiting the template. We will be able to audit and monitor template completion within a practice. We will be able to describe the frequency of template non-completion and subsequent non-enrolment into the study. This will give us some insight into the degree to which selection bias may have occurred. This information will be relayed back to GP practices.
- Patients in both arms of the trial will receive identical study information and questionnaires; minimising the threat of recruitment bias.

Further details about these steps to minimise recruitment and selection bias are provided, where appropriate, in the subsequent sections of the protocol.

***Study participants***

Individuals aged 45 years and older who consult their GP with peripheral joint pain or OA (from a designated set of pre-determined Read codes) will form the sampling base for the trial.

*Inclusion criteria:*

- Aged 45 years and over
- Registered with the participating GP practices during the specified study period of that practice
- Read-coded peripheral joint pain or OA consultation within the specified study period (termed the “index consultation”; may be first, new episode, or ongoing consultation)
- Provided full written informed consent to study participation and to further contact

*Exclusion criteria:*

- Patients who are under active care for or who have a diagnosis of depression and/or an anxiety disorder in the past 12 months
- Vulnerable patients, including any patients on the Quality and Outcomes Framework mental health register, or those who have a diagnosis of dementia or a terminal illness
- Patients who reside in a nursing home
- Red flag pathology – recent trauma associated with significant injury; acute, red, hot swollen joint
- Inflammatory arthropathy, crystal disease, spondyloarthropathy and polymyalgia rheumatica

***Recruitment procedure***

*GP Practices*

Approximately 44 “Research Ready” GP practices that use the EMIS consultation system will be recruited through West Midlands Primary Care Research Network (PCRN) (North Cluster). Information given to practices will be general and they will be told that both arms of the trial will ask some questions that are not unlike those already asked as part of best practice primary care, e.g. assessment of pain intensity and depressive symptoms. Information divulged prior to randomisation will be minimal in order to reduce any bias in the practice deciding to take part as they will be told that they have an equal chance of being allocated to either the control or intervention arm. Practices will be selected on the basis of recent research activity, practice demographics and willingness to participate in the study. The GP practices involved in the study will include those from both urban and rural areas, different socio-economic groups and all sizes of practice (based on patient list size and number of GP partners).

When a practice has agreed to take part in the study, the set-up procedure in the practice will involve downloading anonymised patient information on consultation rates for OA, depression, anxiety, and the comorbid rates of these symptoms. An audit of access to psychological services will also be carried out to ensure that they have the resources to run the study adequately. Details of how practices will be randomised to the trial arms and the post-randomisation procedure are given below.

It is anticipated that the template will be active in each GP practice for approximately three months. GP practices will not all be recruited at once, rather recruitment will take place over a one year period and so the template will not be active in all practices at any one time. A service-level agreement will be ascertained with each GP practice, with a small reimbursement included to pay for practice time

*Patients*

Activation of the screening template will result in a “stamp” on the patient’s electronic record, which will allow our informatics team to establish which patients are eligible for entry into the study. This will also be the case for those templates that have not been completed, either because the GP felt the patient was ineligible or because the GP exited the template without completing it. This will allow us to audit the use of the template and to establish the completion rate, however, we will only be able to describe the characteristics of those patients enrolled into the study and who give consent for their medical records to be reviewed. The patient will be given a postcard during the consultation to make them aware that the research team will contact them via the post.

The PCRN informatics team will visit each practice weekly during their specified study period (approximately three months duration) and download the names and addresses of eligible patients into a secure mailing database. No other information, including the answers to the screening questions, will be downloaded until full written consent for study participation and medical record review is received from the patient (collected in the baseline postal questionnaire – see below). Anonymised aggregate age and gender data for all peripheral joint pain/OA consulters during the one month study period will be downloaded. This will allow us to examine differences between those peripheral joint pain/OA consulters without a completed template, either because the patient was deemed ineligible or for unknown reasons, those who have a completed template but do not consent to take part, and those who have a completed template and consent to take part in the study. This will, to some extent, allow us to determine whether any selection bias has occurred.

All eligible participants (from both arms of the trial) will be sent a study pack in the post that contains:

- a letter from their GP practice introducing the study
- a patient information sheet, which outlines the study and includes instructions of what to do if they do or do not wish to take part
- a baseline questionnaire, including a consent form
- a stamped addressed envelope

As recommended by Eldridge et al (2010), the information provided to patients will be the same for both arms of the trial, helping to eliminate contamination bias [39,40]. Following entry of the names and addresses of eligible patients into a secure mailing database (by a member of the PCRN Informatics team), the study pack will be mailed to eligible patients by an administrative assistant at Arthritis Research UK Primary Care Centre at Keele University who will be blinded to cluster allocation. Letters in the study pack will be on GP practice headed paper and will therefore be specific to each practice that takes part in the study. The patient information sheet and questionnaire will include contact details for the Study Coordinator, who can be contacted should the patient have any further questions about the study. The patient information sheet will also include contact details of their local Patient Advisory Liaison service.

***Randomisation procedure***

GP practices will be randomised in blocks using the balance algorithm for clustered randomised trials [41]. Baseline covariate data will include PCT, general practice list size and the Index of Multiple Deprivation. Practices that rapidly take up the offer of taking part in the study can form a first block, then those that take longer forming a second. The algorithm generates a set of allocations with the smallest imbalance statistic across the two arms. Final allocations will be randomly selected by the independent statistician on the Trial Steering Committee. This information will be passed to the PCRN who will be able to install the appropriate template into each practice and arrange for a GP Research Facilitator to meet with each practice.

***Post-randomisation***

Following randomisation a brief meeting will be held between the GP practice and a GP research facilitator from the PCRN. This will take place for all practices in both arms of the trial and will allow the template to be demonstrated and details of the recommended pathways of care to be disseminated. A laminated crib sheet with the full questions and response options, appropriate to each arm of the trial, will be placed in each consulting room in the GP practices. Details of the information that will be given to both arms is outlined below:

*Intervention arm*

In order to maximise “best practice” in addition to the screening template in the intervention arm, GPs will be referred to and advised to follow NICE recommendations for the holistic assessment and treatment of OA for all patients consulting with peripheral joint pain/OA [20]. GP’s will be recommended to refer to the appropriate NICE guidelines when a patient screens positively for anxiety and/or depression (further details below). A GP Research Facilitator will summarise these recommendations in a PowerPoint presentation and the following 4 papers will be disseminated to the practice (a copy of each paper will be provided for each consulting room):

- NICE clinical guideline 59: “Osteoarthritis. The care and management of osteoarthritis in adults” (quick reference guide) [20]
- NICE clinical guideline 91: “Depression in adults with a chronic physical health problem. Treatment and management” (quick reference guide) [11]
- British Medical Journal summary of new NICE guidelines: “Depression in adults, including those with a chronic physical health problem: summary of NICE guidance” by Pilling et al (2009) [21]
- NICE clinical guideline 113: “Generalised anxiety disorder and panic disorder (with or without agoraphobia) in adults. Management in primary, secondary and community care” (quick reference guide) [42]

Links to the websites where full length version of these guidelines will also be given to the GP practice.

For the case of depression, GP’s will be referred to the “Depression in adults with a chronic physical health problem” guidelines [11], which have a supporting article in the BMJ outlining how to put them into practice [21]. These guidelines suggest 4 steps to follow in the treatment of depression following a positive screen. In brief these are: 1) further questioning to establish the severity of depression; 2) persistent sub threshold depressive symptoms or mild-moderate depression: offering advice on sleep hygiene and actively monitoring depressive symptoms, depending on the severity of symptoms low-intensity psychological intervention or drug treatment may be warranted; 3) non response to initial treatment of severe depression: further treatment options and the possibility of collaborative care; and 4) for severe and complex depression or risk to life: high intensity interventions and medications.

GPs will be advised to follow the stepped care approach for generalised anxiety disorder as outlined in the quick reference clinical guidelines (“Generalised anxiety disorder and panic disorder (with or without agoraphobia) in adults. Management in primary, secondary and community care”) for the treatment of any patients who screen positive for anxiety symptoms [42]. These involve 4 steps: 1) identify, educate and actively monitor; 2) low-intensity psychological intervention; 3) high-intensity psychological intervention or drug treatment; 4) highly specialist treatment.

*Control arm*

In order to keep the control arm as close to “usual care” as possible, GP’s will be advised to follow their usual approach for responding to a patient’s pain intensity rating. Asking a patient the intensity of their pain is common practice in a primary care musculoskeletal pain consultation [20] and therefore should have little effect on the “usual care” that the GP provides. No additional information on OA best practice will be disseminated by the research team.

Having information on pain intensity at consultation in both arms of the trial will provide a further measurement to check that a balance between the intervention and control arms is achieved.

***Activation of the screening template***

Potentially eligible participants will be identified by their GP in the consultation. When the GP enters a peripheral joint pain or OA Read code (from the pre-determined study list) into the electronic patient record then the electronic screening template will be automatically activated. This will only occur for the first time such a Read code is entered for a patient during the study period, ensuring that each patient can only be sampled once. At this stage GPs will apply the exclusion criteria. Activation of the main template will prompt the GP to ask the patient the questions on the template and to record their answers. The consultation will then continue as the GP sees fit; however, details of how to respond to a positive screen for depression and/or anxiety will be reinforced in a post-randomisation meeting (as described above).

During the consultation the GP will provide patients with a short information postcard introducing the study; this is so patients are not surprised when contacted by the research team at later date.

***Informed consent***

In cluster randomised trials participants are usually not asked for their consent to randomisation [43], since cluster randomisation usually occurs before individual participants can be identified [39]. It is accepted in cluster randomised trials that a “guardian” [44] or “gate-keeper” [45] for the cluster provides consent for the individuals in their care to be entered into the trial. In this trial consent will be taken from each GP practice to say that as “guardians” for the patients in their care, they are willing to both enter the trial and to be randomised into either arm of the trial. In cluster randomised trials such as this one, where it would not be feasible to gain individual consent prior to randomisation, it is acceptable to gain individual consent post-randomisation for entry into the study and for follow-up. In this trial participants will be asked to consent to completing postal questionnaires and for their medical records to be accessed. Both control and intervention arms will be given the same information about the trial and although they will not be explicitly informed about the intervention (that has already taken place) selection bias and contamination bias will be reduced [39].

***Mailing procedure***

An illustrated summary of the mailing procedure is provided in Figure 2. All eligible patients will be sent a study pack as outlined above. Those who do not respond within 2 weeks will be contacted again with a postcard reminder. At 4 weeks those who have not responded will be sent a second study pack and reminder letter. At baseline, patients who have still not responded at 8 weeks will be recorded as non-responders and will not be contacted again at any point in the study and their medical records will not be accessed. Participants who respond to the questionnaire but who do not consent to further contact, which will be specified on the consent form as being in 3, 6 and 12 months time, will also be recorded in the database as a non-consenting responder, ensuring that we do not contact them again in the future (this may occur by returning a completed or blank questionnaire or verbally by telephone conversation).

*Follow-up*

Participants who return a questionnaire and consent to participate at baseline will be sent a follow-up questionnaire at 3, 6 and 12 months following their index consultation. In order to maximise participation a short questionnaire, including only minimal data collection items (outlined below), will be sent to participants who have not responded to the full postal questionnaire at 6 weeks. At 6 and 12 month follow-up points an additional telephone call will be used at 8 weeks (for those participants who provided a telephone number in their baseline questionnaire) to attempt to contact non-responders in order to get information on their pain intensity at that time. Three telephone call attempts will be made to contact each participant. The mailing procedure is outline in Figure 2.

**Figure 2:** Mailing procedure


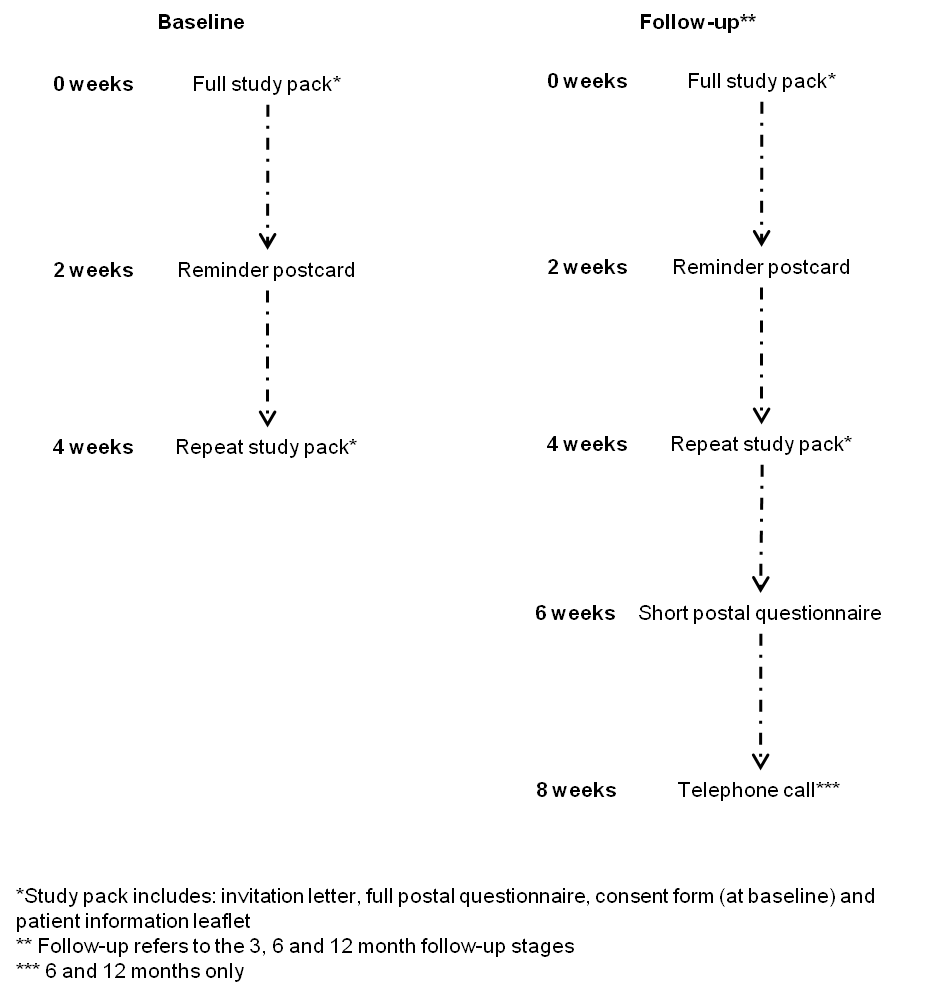


***Data collection***

Data collection will take three forms: screening template in the GP consultation, postal questionnaire and medical record review.

1. *Electronic screening template*

The screening template consists of five and one question(s) for the intervention and control arms, respectively. The content for both arms is summarised in Table 1. Appendix 1 gives full details of the screening template for both arms of the trial, including the response options. All responses will be recorded on the pop-up template during the consultation.

*Intervention arm*

In order to screen for depression two items previously validated in primary care populations [12] will be used. These questions are adapted from the PHQ-2 [46] and are routinely used as part of the General Practice Contract for patients with CHD and diabetes and are also recommended by the recent guidelines for comorbid depression with a chronic physical health problem [11]. Anxiety will be screened for using two items adapted from the GAD-2 [26]. Both the PHQ-2 and GAD-2 have been shown to be reliable and valid ultra-short screening tools for depression and anxiety in a general population setting [47]. In both instances the adaptations from the original 2-item depression and anxiety tools refers to a simplified response of “yes” or “no” to having experienced such symptoms in the past month. The intervention arm will also be asked to rate their current pain on a numerical rating scale from 0 (“no pain”) to 10 (“pain as bad as it could be”). Patients will be considered as having screened positive for depression if they answer “yes” to either of the two depression questions. Similarly, answering “yes” to at least 1 of the 2 anxiety questions will result in a positive screen for anxiety.

*Control arm*

The template for the control arm will prompt GPs to ask patients to rate their current pain on a numerical rating scale from 0 (“no pain”) to 10 (“pain as bad as it could be”). A question typical of those routinely asked in an OA consultation.

**Table 1:** Summary of screening template content

|  | **Conceptual domain** | **Operational definition** | **Empirical measure** | **Number of items** |
| --- | --- | --- | --- | --- |
| **Intervention arm** | Pain intensity | Current pain intensity (of the index pain, at the time of the index consultation) | 0-10 NRS (48) | 1 |
|  | Depression | Low mood, anhedonia in the past month | Depression screening question for primary care (12) | 2 |
|  | Anxiety | Nervousness and worrying in the past month | Modified GAD-2 (26) | 2 |
| **Control Arm** | Pain intensity | Current pain intensity (of the index pain, at the time of the index consultation) | 0-10 NRS (48) | 1 |
| NRS – numerical rating scale | | | | |

*2a. Full postal Questionnaire*

The postal questionnaires are designed to collect information on descriptive characteristics of the participants, in-depth depression and anxiety measures, pain-related characteristics and detailed data collection of our outcome measures. The items to be included in each of the four postal questionnaires (baseline, 3, 6 and 12 months) are summarised in Table 2. Further details of the outcome measures of interest are provided below.

*Outcome measures*

1. *Primary outcomes*

The primary outcome measure of the study will be index pain severity across the study time period. The Chronic Pain Grade [48] will be included in each of the postal questionnaires, this includes the question “How would you rate your pain on a 0-10 scale at the **present time** where 0 is “no pain” and 10 is “pain as bad as it could be”?”. This question is also part of the consultation screening template for both arms of the study.

1. *Secondary outcomes*

Pain interference with daily activities will be assessed on a numerical rating scale of 0-10. The numerical rating scales for pain intensity and pain interference form part of the Chronic Pain Grade [48], a 7-item scale from which composite pain intensity and interference scores will be generated. A further secondary outcome measure is global function, as assessed by the SF-12 [49]. Physical function relating specifically to knee and/or hip pain will be assessed using the short form WOMAC function subscale (8 items) and two stem questions to determine which participants have knee and/or hip pain [50]. Detailed assessment of depressive and anxiety symptoms will be collected using the PHQ-8 and GAD-7, respectively. The PHQ-8 is derived from the PHQ-9; a validated 9-item measure of depression designed to both diagnose and assess the severity of depression [51,52]. The GAD-7 was established using both DSM-IV criteria for general anxiety disorder and items from previous existing anxiety scales [53]. As with the PHQ-8, the GAD-7 enquires about symptoms experienced over the last two weeks. The first two items on the PHQ-9 and GAD-7 comprise the PHQ-2 and GAD-2, respectively, which are used to screen for depressive and anxiety symptoms in the intervention arm of this trial. Additional items will be included in the questionnaire to gather further information about participants’ index pain symptoms, other sites of pain, pain catastrophising scale [54], general medical health (including comorbidities and medication use) and demographic details.

1. *Health economics outcomes*

Costs: Health sector costs will include primary and secondary care contacts, investigations, medication and contacts with other health care professionals such as physiotherapists and community psychiatric nurses. Primary health care resource use (GP and practice nurse consultations/home visits) and medications will be collected from a review of primary care medical records, covering the full 12 month follow-up period for all study participants. Information on secondary care attendances and patient borne costs will be gathered using the postal self-report questionnaires. Questions on patients’ personal expenditure will concentrate on private health care use and over-the-counter treatments. Productivity costs will be calculated using data collected on occupation and days off work due to illness. Unit costs will be obtained from standard sources and health care providers. Due to the nature of the study, generic health care resource use will be collected (i.e. not specific to the treatment of OA). Information on resource use from the self-report questionnaires will be collected at 6 months and 12 months, with each questionnaire covering a 6 month recall period.

Quality of life measures: All patients will be asked to complete the 5-level version of the EuroQoL-5D (EQ-5D) [55] questionnaire at baseline, 3 months, 6 month and 12 months in order that quality-adjusted life years (QALYs) over the 12 month time period can be calculated for each study participant. The QALYs combine information on health-related quality of life and survival. The SF-12 will also be included as a measure of generic health-related quality of life, thus allowing an alternative method of calculating QALYs using the SF-6D in a sensitivity analysis.

The conceptual domains, operational definitions and empirical measures of each of the items included in the postal questionnaires are detailed in Table 2. This table also highlights in which of the questionnaires (baseline, 3, 6 or 12 months) the item is included.

**Table 2:** Summary of full postal questionnaire content

| **Conceptual domain** | **Operational definition** | **Empirical measure** | **Number of items** | **Time** |
| --- | --- | --- | --- | --- |
| **Primary Outcomes** |  |  |  |  |
| Pain intensity | Current intensity of the index pain | NRS 0-10 * | 1 | B, 3FU, 6FU, 12FU |
| **Secondary Outcomes** | |  |  |  |
| Disability | Composite characteristic pain interference with daily activities, recreation and work during the past 3 months. | Number of days (1 item) and NRS 0-10 (3 items provide composite disability score of 0-100)* | 4 | B, 3FU, 6FU, 12FU |
| Characteristic pain intensity | Composite current and past and average pain intensity during the past 3 months. | NRS 0-10 (3 items provide a composite characteristic pain intensity score of 0-100)* | 3 | B, 3FU, 6FU, 12FU |
| Index pain location | Site of index pain complaint | Choice of anatomical site | 1 | B |
| Nature of onset | Traumatic onset | Yes / no / unsure | 1 | B |
| Episode duration | Time since last whole month free from this pain | Episode duration (56) | 1 | B, 3FU. 6FU, 12FU |
| Days in pain | Days in past 6 months with index pain | No days / 1-30 days / 31-89 days / 90+ days | 1 | B, 6FU, 12FU |
| Multiple site / chronic widespread pain | Pain in sites other than the index site over the last month. Manchester definition of widespread pain; Keele definition of number of pain sites; chronicity of pain; pain in the knee or hip | Yes/no; Body manikin (57,58) | 4 | B, 3FU, 6FU, 12FU |
| Pain consultation | First consultation for this pain complaint | Yes / no | 1 | B |
| Pain improvement | Improvement in pain at index pain site | 6 point Likert scale | 1 | 3FU, 6FU, 12FU |
| Further pain consultation | Consulted since index consultation with the same pain site | Yes / no | 1 | 3FU, 6FU, 12FU |
| Satisfaction with consultation | Satisfaction with questions asked during consultation | Yes/no/unsure; details; list of items asked | 4 | B |
| Physical function related to knee and/or hip pain | Degree of difficulty with 8 daily activities | Short form WOMAC function subscale (50) | 8 | B, 3FU, 6FU, 12FU |
| Catastrophic coping | Catastrophic coping relating to index pain | Pain catastrophising scale (54) | 9 | B |
| Anxiety | Generalised anxiety symptoms in past 2 weeks | GAD-7 (53) | 7 | B, 3FU, 6FU, 12FU |
| Depression | Depressive symptoms in past 2 weeks | PHQ-8 (59) | 8 | B, 3FU, 6FU 12FU |
| Discussion of mood in consultation | Did doctor ask about mood during index consultation | Yes / no / unsure | 1 | B |
| Diagnosis of depression | Ever diagnosed by doctor as depressed | Yes / no / unsure | 1 | B |
| Diagnosis of anxiety | Ever diagnosed by doctor with an anxiety disorder | Yes / no / unsure | 1 | B |
| **Quality of life measures** | |  |  |  |
| General health | Mental and physical well-being | SF-12 (49) | 12 | B, 3FU, 6FU 12FU |
| General health | Utility-based quality of life | EuroQol-5D (55) | 5 | B, 3FU, 6FU 12FU |
| Comorbidities | Ever been diagnosed with any from a list of possible comorbidities | Yes |  | B |
| Fractures | Ever fractured hip/wrist/other bones |  |  | B |
| Falls | Falls in the past 12 months | Yes/No | 1 | B |
| **Healthcare costs** |  |  |  |  |
| Medication usage | Select from list of analgesic and psychiatric medicines that currently taking |  |  | B |
| Health care resource use | Use of non-primary care health care resources | Yes/No and details of resource use if Yes | 3 | B, 6FU, 12FU |
| OTC medication | OTC medication expenditure | Details of OTC medications |  | 6FU, 12FU |
| **Miscellaneous** |  |  |  |  |
| Age | Age at index consultation | Date of birth | 1 | B, 3FU, 6FU, 12FU |
| Sex | Sex | Male / Female | 1 | B, 3FU, 6FU, 12FU |
| Employment status and absence from work | Employment status at time of questionnaire and days of work absence | Yes/No and details | 10 | B, 6FU, 12FU |
| Socioeconomic status | Occupational class based on individual (i) current or (ii) most recent job title | Job title – categorised as manual / non-manual (60,61) | 2 | B |
| Living arrangement | Live alone | Yes / No | 1 | B |
| Marital status | Marital status at time of Baseline Questionnaire | Married / Single / Divorced / Widowed / Separated / Cohabiting | 1 | B |
| Social support | Availability of instrumental and emotional support (yes/no/no need) | Single items (62) | 2 | B, 6 FU, 12 FU |
| Obesity | Body Mass Index | Height (m / ft, in), weight (kg / st, lb) | 2 | B |
| B – baseline; FU – follow-up (in months); OTC – over the counter; NRS – numerical rating scale; * 7 items form the Chronic Pain Grade (48). | | | | |

*2b. Short postal questionnaire*

This questionnaire will contain a minimum set of data that we are interested in, namely: Chronic Pain Grade and EQ-5D (summarised in Table 3).

**Table 3:** Summary of short postal questionnaire content

| **Conceptual domain** | **Operational definition** | **Empirical measure** | **Number of items** | **Time** |
| --- | --- | --- | --- | --- |
| Index global severity | Composite characteristic pain intensity, interference with activities, and disability days (of the index pain, in the month preceding index consultation) | Chronic Pain Grade (48)* | 7 | 3FU, 6FU, 12FU |
| General health | Utility-based quality of life | EuroQol-5D (55) | 5 | 3FU, 6FU 12FU |
| Age | Age at index consultation | Date of birth | 1 | 3FU, 6FU, 12FU |
| Sex | Sex | Male / Female | 1 | 3FU, 6FU, 12FU |
| FU – follow-up (in months); * Chronic pain grade includes primary outcome measure of current pain intensity | | | | |

*2c. Telephone questionnaire*

At each of the 6 and 12 month follow-up timepoints we will attempt to contact by telephone participants who do not respond to either the full or short questionnaires. Participants will be asked the brief questions regarding the intensity of the pain in their index pain site and how much this interferes with their daily activities. They will be asked to rate these on a numerical rating scale of 0-10 (summarised in Table 4).

**Table 4:** Summary of telephone questionnaire content

| **Conceptual domain** | **Operational definition** | **Empirical measure** | **Number of items** | **Time** |
| --- | --- | --- | --- | --- |
| Pain intensity and interference | Pain intensity and interference with activities | NRS (0-10) | 2 | 6FU, 12FU |
| Age | Age at index consultation | Date of birth | 1 | 6FU, 12FU |
| FU – follow-up (in months); NRS – numerical rating scale | | | | |

1. *Medical record review*

A medical record review will take place for all participants who have given their consent (at the time of the baseline questionnaire) to have their medical records reviewed (summarised in Table 5). This will allow us to download their data from the consultation screening template. Date of consultation, general practice and index Read code information will also be collected at this time. After the 12 month follow-up period a more detailed review will be carried out to enable us to assess prescriptions, referrals for further treatment, and number of consultations and whether these are for OA or mood problems. It will also allow us to determine whether there had been any previous diagnosis or treatment for depressive or anxiety symptoms. This data will provide primary health care resource use for the health economic analysis. The information collected on the treatment paths taken for patients who have a completed template will be fed back to practices following the 12 month record review, allowing practices in the intervention arm to see how closely they have followed recommended NICE guidelines. This information will also be of interest to GP practices in the control arm.

**Table 5:** Summary of medical record review

| Item | **Time** |
| --- | --- |
| Content of screening template | B |
| Prescriptions | 12FU |
| Referrals | 12FU |
| Number of OA / mood problems consultations | 12FU |
| B – baseline; FU – follow-up (in months) | |

***Study administration and data storage***

The Study Coordinator will oversee the day to day running of the study. Members of the PCRN will download details of patients who have a completed template (name, address, date of birth and EMIS identification code) on a weekly basis from each practice. Two secure databases will be designed specifically for use in this study. One will be a mailing database where all details downloaded from practice databases will be stored. A unique study number will be applied to each potential participant. The Research Support Coordinator will arrange mailings of the study pack, following the mailing procedure outlined above. On return of a completed questionnaire details will be entered into the mailing database to ensure no unnecessary reminders are sent to participants. Details of informed consent will be stored on the mailing database. Following the reminder period of the mailing procedure, eligible patients who do not respond to the baseline questionnaire will be recorded as non-responders in the mailing database and will not be contacted again at any stage of the study.

The second database will be used to store all data collected during the study. In this database participants will only be identified by study number. Data will be entered into both databases by trained members of the administration team who will be blinded to cluster allocation. Access to the databases will be restricted to those members of the team that are working on this study. The coding schedule for the study questionnaires will be used to inform database design and to facilitate data entry. The data entry of a randomly selected 1 in 10 questionnaires will be checked by a member of the administration team who has not been inputting data from this study and who is blinded to cluster allocation. Details of data entry accuracy will be kept by the Research Support Coordinator and the study statistician and reported to Trial Steering Committee and Date Monitoring Committee. For those participants who agree to a medical record review, data from their completed screening template will be downloaded.

Throughout the entire mailing process, fortnightly checks will be carried out at each practice, by a member of the PCRN, to determine any patient deaths and departures. This will ensure we do not inappropriately contact any patients throughout the time period of the study. The name and address details of non-respondents will be erased after a reasonable amount of time. The NHS tracing system will be used to search for any non-responders to ensure we have the correct postal address.

On receipt of a completed questionnaire it will be checked for missing questions or an incomplete consent form, if it seems that any pages have been skipped over by accident then these pages will be photocopied and sent, along with an accompanying letter and stamped addressed envelope, to the participant for completion. If answers in the questionnaire are missing or appear to be ambiguous they will be coded as such in the database.

Regular meetings will be held with the Research Support Coordinator, PCRN and Study Coordinator to resolve any arising issues with regard to the everyday running of the study. Requests for data must be made in writing along with a detailed analysis plan to the data custodian for the study. Questionnaires and study consent sheets will be stored securely in separate locations to ensure participant confidentiality.

The trial will be conducted in compliance with this protocol and Good Clinical Practice guidelines. The Trial Steering Committee and Data Monitoring Committee will monitor the progress of the trial and, following their terms of reference, will intervene where they deem necessary.

***Blinding***

The Principal Investigator, Trial Statistician and members of the administration team, who input data from the study questionnaires, as well as the GPs, will be blinded to cluster allocation. The Trial Steering Committee and Data Monitoring Committee will also be blinded to cluster allocation unless it becomes absolutely necessary to reveal allocation.

## Sample size

In order to use the information recorded at all the time points we have considered the sample size needed to estimate the time-averaged difference in the pain response between the two arms. Diggle et al (2002) give the number of subjects needed per arm as M=2(z_α_ + z_β_)^2^{1+ (n-1)ρ}/(nΔ^2^), using a significance level of α, power 1-β, Δ= smallest meaningful difference in standard deviation units, n=number of repeated measures [63]. Given that the mean difference is expected to be zero at baseline, we will think of the effect size as an average of 0.2 over the three follow up time points. Using ρ=0.5 as an estimate of the autocorrelation, n=3, Δ=0.2, α=0.05, β=0.10, gives 350 per arm. To adjust for the clustering of the practices we modify this figure by the design effect (1+(m-1)*icc) for an average cluster size m. Using an icc=0.03, average cluster size of 30, the modified figure is 1309 leading to 44 practices to be recruited. Erring on the side of caution and adjusting for 25% non-response to the baseline questionnaire, 1745 patients will need to be screened and contacted; equivalent to approximately 40 patients per practice. Evidence suggests a consultation rate for OA and peripheral joint pain is at least 15 per month per typical GP practice, suggesting that the template will need to be active for approximately three months at each GP practice. If this poses logistical challenges, reducing the power to an acceptable 80% would result in 980 patients needed to be recruited from 34 practices with clusters of 30 patients (or 1307 to be screened and contacted across the 34 practices to allow for 25% non-response). The run-in period will further inform the estimate of non-response to the baseline questionnaire and if nescessary allow us modify the adjusted sample size needed.

# *Statistical analysis*

Both the primary and secondary analysis will be by intention to treat and appropriate multiple imputation techniques will be used to account for missing data. The primary outcome, the current pain intensity component (0-10 numerical rating scale) of the Chronic Pain Grade [48], will be treated as interval in nature. Multilevel linear mixed modelling will be used to account for the clustered nature of the data. Using treatment by group interaction terms, differences in the mean response profiles will be investigated and quantified using appropriate 95% confidence intervals for the differences between intercept and gradient estimates.

Standard goodness of fit tests will be applied and if the distribution of the residuals is markedly non-normal, bootstrapping will be considered. Time varying covariates can also be accommodated within the multilevel framework.

***Cost-utility analysis***

The health economic analysis will determine the cost-utility of screening patients for anxiety and depression in OA compared with a standard consultation without screening. A cost-consequence analysis will initially be reported, describing all the important results relating to costs and consequences. Subsequently, an incremental cost-utility analysis will also be undertaken using patient responses to the EQ-5D questionnaire, to calculate the cost per additional QALY gained. The base case cost analysis will adopt a National Health Service (NHS) and personal social services (PSS) perspective [64]. A broader costing perspective will be considered in a sensitivity analysis, taking into account NHS/PSS costs, patients’ personal expenditure and costs associated with work loss. The data for costs is likely to have a skewed distribution therefore the plan is to explore the nature of the distribution of costs. If the data is not normally distributed, the non parametric comparison of means (e.g. bootstrapping) will be undertaken. Where differences in baseline characteristics occur between trial arms, for example in baseline EQ-5D score, the analysis will control for these baseline differences using regression-based adjustment. Efforts will be made to minimise the problem of missing data, but if this does pose a problem at the analysis stage then multiple imputation techniques will be used. The robustness of the results will be explored using sensitivity analysis. This will explore uncertainties in the trial based data itself, the methods employed to analyse the data and the generalisability of the results to other settings. Uncertainty in the confidence to be placed on the results of the economic analysis will be explored by estimating cost effectiveness acceptability curves. These plot the probability that the intervention is cost effective against threshold values for cost effectiveness.

***Run-in period***

An internal pilot of the trial protocol will take place in four practices, two in each arm. This “run-in” period will be used to ensure that our methods and key assumptions outlined in this protocol work successfully and that the day-to-day administration of the trial runs smoothly. Particular attention will be paid to the recruitment of GP practices, completion rate of the screening template, response rate to, and completion rate of the baseline postal questionnaire and the consent rate to further contact and medical record review. This phase of the trial will run until recruitment at these four practices is complete, which we anticipate to take three months. The template will not be activated in any further practices until this run-in period is complete. Data collected during this phase of the trial will be included in final data analysis as no interim analysis will be carried out with this data. Data from this phase of the trial will only be excluded from the final analysis if any substantial amendments to the current protocol have to be made.

**Timescale**

The timing of the study milestones is illustrated in Figure 3.

# Closing remarks

The implementation of this cluster randomised trial will allow us to investigate the effect of prompting GPs to ask about concurrent anxiety and depressive symptoms during a peripheral joint pain/OA consultation screening. A previous observational study within our centre has demonstrated the feasibility of GPs carrying out depression screening in the OA consultation and shown our ability to subsequently recruit and follow-up patients [23]. The run-in period at the beginning of the trial with four GP practices will allow us to test our assumptions and processes before moving on to the main trial.

**Amendment to POST Study Protocol_V2.0_24.5.11:**

**A nested substudy of general practitioners’ experiences of participating in POST**

This component of the trial is intended to explore individual GP’s experience of participating in the POST study. The objective of this component is to identify barriers and facilitators related to screening OA/joint pain patients in both arms of the trial and to explore the experience and perceived value of including questions about anxiety and depressive symptoms. It will also explore how GPs have engaged with the trial and in the use of the screening template during their consultations, and whether there are differences between the two arms of the trial. There will be two methods used to explore GP’s experience of participating in POST, firstly a brief questionnaire that will be distributed to all GPs at participating GP practices, and secondly, a semi-structured interview with a sub-group of participating GPs. Both of these methods are outlined below.

1. **GP questionnaire**

**Study participants:** All general practitioners at the general practices taking part in POST

**Method:** Post-intervention self-complete questionnaire. This is a brief 7 item questionnaire (Table 6) that will gather information on the GPs opinions on participating in POST. A similar questionnaire was used to gather feedback from GPs involved in a previous research study that involved the use of a screening template. The same questionnaire will be completed by GPs in both arms of the trial. All information and instructions are included in the questionnaire. As part of their participation in the trial GPs are made aware that we will ask them for feedback at the end of their study period and therefore a separate GP information leaflet and cover letter is not required. The questionnaires will not be addressed to specific GPs, instead copies of the questionnaire will be given to the Practice Manager at the end of the GP practice’s study period (approximately 3 months) and he/she will ask the GPs at their practice to complete the questionnaire. Completed questionnaires will be returned via the Practice Manager to this Research Centre in the stamped address envelope provided.

The back page of the questionnaire explains that we may wish to contact the GP to discuss some of their responses further and asks the GP to provide their name and contact details. When a questionnaire is returned to the centre the information will be entered into a database and at that stage a study number will be assigned to the questionnaire. The back page of the questionnaire will be removed from the questionnaire and stored separately in a locked filing cabinet in the administration department of this Research Centre. The information given in the questionnaire will be useful to us regardless of whether contact information is provided; however, having contact information for some GPs will help us to target our sampling for qualitative interviews (as outlined below). For those GP participants who later take part in a semi-structured interview, their questionnaire responses will be used to inform that interview.

**Analysis:** Simple descriptive characteristics.

**Table 6:** Content of GP’s experience of participating in POST questionnaire

| **Item Number** | **Item Description** | **Item response** |
| --- | --- | --- |
| 1 | Impact on consultation time | 5 point Likert scale |
| 2 | Amount of additional time | 7 options plus free text option if appropriate |
| 3 | Impact on doctor-patient communication | 5 point Likert scale plus additional 4 point Likert scale where there was a impact |
| 4 | Effect on patient management | 5 point Likert scale |
| 5 | Reasons for excluding patients | free text |
| 6 | Ease of incorporating screening questions | 4 point Likert scale |
| 7 | Additional comments | Free text |
|  | Name |  |
|  | Contact details |  |

1. **Semi-structured GP interviews**

This component of the study involves semi-structured interviews with a sub-group of participating GPs. This interview schedule, which is based around the work of Coventry et al (2011) for screening for depressive symptoms in primary care patients with a long term health problem [65], is designed to complement the GP questionnaire, the responses to which will also contribute to the basis of the interview. GPs in the control arm were not asked to screen for depressive and anxiety symptoms, and therefore questions relating to the impact of screening for these symptoms on consultation time, doctor-patient communication and GP engagement will be limited to GPs in the intervention arm of the trial. However, those in the control arm will be asked their general views on the frequency and degree of presentation of such symptoms amongst their patients. In addition, both arms asked the question regarding pain intensity and so questions regarding the impact that this had will be included in the interview schedule for both arms. GPs from both arms of the trial will also be asked about the acceptability of screening in general for OA/joint pain patients and in how they have engaged with the study. The interview schedules are considered as topic guides for the interviews.

**Study participants:** It is anticipated that up to 40 interviews may be conducted, 20 from each arm. However, interviewing will continue until saturation is reached, i.e. no new themes are emerging from the process of on-going analysis. GPs will be sampled from those who have given their contact details in the GP questionnaire, ideally these will include GPs from practices that have screened the target number of patients, exceeded their screening target and screened less than target.

**Method:** Semi-structured interviews (based on the interview schedules (summarised in Table 7)), estimated to last for approximately 30 minutes, will be conducted with GPs who have consented to take part. Those GPs who have provided their contact details in the questionnaire will be provided with an information letter. There are two versions of this letter, one for GPs in each of the trial arms. The letter contains all the information necessary to make an informed decision on whether to continue to participate. A follow-up telephone call will be made in order to see if GPs are indeed happy to take part and to arrange a convenient time for the interview to take place. Informed consent will be taken at the time of the interview by the trained interviewer and all interviews will be audio recorded.

**Analysis:** The audio recordings will be fully transcribed in an anonymised format. Content analysis will be performed with the specific focus on the research questions as outlined in the interview schedule. As codes emerge, data will be checked for additional confirmatory or challenging evidence within and between individual interviews, and across the two groups in line with basic principles of qualitative data analysis [66]. Where possible, data from the questionnaires will be combined those from the interviews in line with a mixed methods approach [67].

**Ethics:** Anonymity is the principal ethical challenge. To address this, participants will be given a unique ID number and pseudonym. Identifiable names of people, places or institutions will be removed from each transcript. Digital recordings will be held on a dedicated secure University server, and will only be accessible to those directly involved in the study. All quotations in reports, publications, and presentations will be presented in an anonymous format.

**Table 7:** Outline of interview schedule for GPs

| **Domains** | **Intervention arm (yes/no)** | **Control arm (yes/no)** |
| --- | --- | --- |
| General patient population including psychological problems | Yes | Yes |
| Views of psychological problems in joint pain/OA | Yes | Yes |
| Presentation and detection of psychological symptoms | Yes | Yes |
| Management | Yes | Yes |
| Communication | Yes | Yes |
| Training and development | Yes | Yes |
| Taking part in POST | Yes | Yes |
| Follow-up | Yes | Yes |
| GP practice | Yes | Yes |
| Ease of asking questions | Yes | No |
| Differences between GAD & PHQ | Yes | No |
| Usefulness of screening template | Yes | No |
| Patients included/excluded | Yes | No |

**Timeline**

The screening template is currently being turned off at the 8 GP practices involved in block 1 of the trial. 5 GP practices in block 2 are currently active. Ideally the GP questionnaires will be distributed to GPs as close to the end of their study period as possible, so that recall inaccuracy and bias are limited. The semi-structured interviews can begin to take place with GPs whose study period has finished any time after ethics approval has been granted. Due to the nature of GP practices participating in blocks and the study actively recruiting until approximately November 2012, these can take place over a period of time, depending on the availability of the interviewer and GPs. Again, ideally they would be conducted soon after a GP practice’s template has been deactivated.

# Figure 3: Study timescale

|  | **2011** | | | | | | | | | | | | **2012** | | | | | | | | | | | | **2013** | | | | | | | | | | | | **2014** | | | | | | |
| --- | --- | --- | --- | --- | --- | --- | --- | --- | --- | --- | --- | --- | --- | --- | --- | --- | --- | --- | --- | --- | --- | --- | --- | --- | --- | --- | --- | --- | --- | --- | --- | --- | --- | --- | --- | --- | --- | --- | --- | --- | --- | --- | --- |
|  | **J** | **F** | **M** | **A** | **M** | **J** | **J** | **A** | **S** | **O** | **N** | **D** | **J** | **F** | **M** | **A** | **M** | **J** | **J** | **A** | **S** | **O** | **N** | **D** | **J** | **F** | **M** | **A** | **M** | **J** | **J** | **A** | **S** | **O** | **N** | **D** | **J** | **F** | **M** | **A** | **M** | **J** | **J** |
| Ethical approval |  |  |  |  |  |  |  |  |  |  |  |  |  |  |  |  |  |  |  |  |  |  |  |  |  |  |  |  |  |  |  |  |  |  |  |  |  |  |  |  |  |  |  |
| GP practice recruitment |  |  |  |  |  |  |  |  |  |  |  |  |  |  |  |  |  |  |  |  |  |  |  |  |  |  |  |  |  |  |  |  |  |  |  |  |  |  |  |  |  |  |  |
| Randomisation of GP practices |  |  |  |  |  |  |  |  |  |  |  |  |  |  |  |  |  |  |  |  |  |  |  |  |  |  |  |  |  |  |  |  |  |  |  |  |  |  |  |  |  |  |  |
| Patient recruitment |  |  |  |  |  |  |  |  |  |  |  |  |  |  |  |  |  |  |  |  |  |  |  |  |  |  |  |  |  |  |  |  |  |  |  |  |  |  |  |  |  |  |  |
| Run-in period |  |  |  |  |  |  |  |  |  |  |  |  |  |  |  |  |  |  |  |  |  |  |  |  |  |  |  |  |  |  |  |  |  |  |  |  |  |  |  |  |  |  |  |
| Follow-up |  |  |  |  |  |  |  |  |  |  |  |  |  |  |  |  |  |  |  |  |  |  |  |  |  |  |  |  |  |  |  |  |  |  |  |  |  |  |  |  |  |  |  |
| Medical record download |  |  |  |  |  |  |  |  |  |  |  |  |  |  |  |  |  |  |  |  |  |  |  |  |  |  |  |  |  |  |  |  |  |  |  |  |  |  |  |  |  |  |  |
| Data entry |  |  |  |  |  |  |  |  |  |  |  |  |  |  |  |  |  |  |  |  |  |  |  |  |  |  |  |  |  |  |  |  |  |  |  |  |  |  |  |  |  |  |  |
| Data cleaning |  |  |  |  |  |  |  |  |  |  |  |  |  |  |  |  |  |  |  |  |  |  |  |  |  |  |  |  |  |  |  |  |  |  |  |  |  |  |  |  |  |  |  |
| Statistical analysis |  |  |  |  |  |  |  |  |  |  |  |  |  |  |  |  |  |  |  |  |  |  |  |  |  |  |  |  |  |  |  |  |  |  |  |  |  |  |  |  |  |  |  |
| Writing-up |  |  |  |  |  |  |  |  |  |  |  |  |  |  |  |  |  |  |  |  |  |  |  |  |  |  |  |  |  |  |  |  |  |  |  |  |  |  |  |  |  |  |  |
| **GP’s experience of participating in POST** |  |  |  |  |  |  |  |  |  |  |  |  |  |  |  |  |  |  |  |  |  |  |  |  |  |  |  |  |  |  |  |  |  |  |  |  |  |  |  |  |  |  |  |
| Ethics and R&D amendment |  |  |  |  |  |  |  |  |  |  |  |  |  |  |  |  |  |  |  |  |  |  |  |  |  |  |  |  |  |  |  |  |  |  |  |  |  |  |  |  |  |  |  |
| GP questionnaire |  |  |  |  |  |  |  |  |  |  |  |  |  |  |  |  |  |  |  |  |  |  |  |  |  |  |  |  |  |  |  |  |  |  |  |  |  |  |  |  |  |  |  |
| GP interviews |  |  |  |  |  |  |  |  |  |  |  |  |  |  |  |  |  |  |  |  |  |  |  |  |  |  |  |  |  |  |  |  |  |  |  |  |  |  |  |  |  |  |  |
| Data entry / cleaning (including transcription) |  |  |  |  |  |  |  |  |  |  |  |  |  |  |  |  |  |  |  |  |  |  |  |  |  |  |  |  |  |  |  |  |  |  |  |  |  |  |  |  |  |  |  |
| Analysis |  |  |  |  |  |  |  |  |  |  |  |  |  |  |  |  |  |  |  |  |  |  |  |  |  |  |  |  |  |  |  |  |  |  |  |  |  |  |  |  |  |  |  |
| Writing-up |  |  |  |  |  |  |  |  |  |  |  |  |  |  |  |  |  |  |  |  |  |  |  |  |  |  |  |  |  |  |  |  |  |  |  |  |  |  |  |  |  |  |  |
|  |  |  |  |  |  |  |  |  |  |  |  |  |  |  |  |  |  |  |  |  |  |  |  |  |  |  |  |  |  |  |  |  |  |  |  |  |  |  |  |  |  |  |  |

**References**

(1) Arthritis Research UK. Osteoarthritis: An Information Booklet. 2004.

(2) Porcheret M, Jordan K, Croft P, Primary Care Rheumatology Society. Treatment of knee pain in older adults in primary care: development of an evidence-based model of care. Rheumatology (Oxford) 2007 Apr;46(4):638-648.

(3) Axford J, Heron C, Ross F, Victor CR. Management of knee osteoarthritis in primary care: pain and depression are the major obstacles. J Psychosom Res 2008 May;64(5):461-467.

(4) Gureje O, Von Korff M, Simon GE, Gater R. Persistent pain and well-being: a World Health Organization Study in Primary Care. JAMA 1998 Jul 8;280(2):147-151.

(5) Arnow BA, Hunkeler EM, Blasey CM, Lee J, Constantino MJ, Fireman B, et al. Comorbid depression, chronic pain, and disability in primary care. Psychosom Med 2006 Mar-Apr;68(2):262-268.

(6) Rosemann T, Backenstrass M, Joest K, Rosemann A, Szecsenyi J, Laux G. Predictors of depression in a sample of 1,021 primary care patients with osteoarthritis. Arthritis Rheum 2007 Apr 15;57(3):415-422.

(7) Lin EH, Katon W, Von Korff M, Tang L, Williams JW,Jr, Kroenke K, et al. Effect of improving depression care on pain and functional outcomes among older adults with arthritis: a randomized controlled trial. JAMA 2003 Nov 12;290(18):2428-2429.

(8) Lin EH, Tang L, Katon W, Hegel MT, Sullivan MD, Unutzer J. Arthritis pain and disability: response to collaborative depression care. Gen Hosp Psychiatry 2006 Nov-Dec;28(6):482-486.

(9) Bair MJ, Robinson RL, Katon W, Kroenke K. Depression and pain comorbidity: a literature review. Arch Intern Med 2003 Nov 10;163(20):2433-2445.

(10) Freeling P, Rao BM, Paykel ES, Sireling LI, Burton RH. Unrecognised depression in general practice. Br Med J (Clin Res Ed) 1985 Jun 22;290(6485):1880-1883.

(11) National Institute for Health and Clinical Excellence. The treatment and management of depression in adults with chronic physical health problems. 2009;Clinical guidelines CG91.

(12) Arroll B, Khin N, Kerse N. Screening for depression in primary care with two verbally asked questions: cross sectional study. BMJ 2003 Nov 15;327(7424):1144-1146.

(13) Katon W, Unutzer J, Fan MY, Williams JW,Jr, Schoenbaum M, Lin EH, et al. Cost-effectiveness and net benefit of enhanced treatment of depression for older adults with diabetes and depression. Diabetes Care 2006 Feb;29(2):265-270.

(14) Gilbody S, House AO, Sheldon TA. Screening and case finding instruments for depression. Cochrane Database Syst Rev 2005 Oct 19;(4)(4):CD002792.

(15) Gilbody S, Sheldon T, Wessely S. Should we screen for depression? BMJ 2006 Apr 29;332(7548):1027-1030.

(16) Katon W, Lin EH, Kroenke K. The association of depression and anxiety with medical symptom burden in patients with chronic medical illness. Gen Hosp Psychiatry 2007 Mar-Apr;29(2):147-155.

(17) Nimalasuriya K, Compton MT, Guillory VJ. Screening adults for depression in primary care: A position statement of the American College of Preventive Medicine. J Fam Pract 2009 Oct;58(10):535-538.

(18) Whooley MA, Avins AL, Miranda J, Browner WS. Case-finding instruments for depression. Two questions are as good as many. J Gen Intern Med 1997 Jul;12(7):439-445.

(19) U.S. Preventive Services Task Force. Screening for depression: recommendations and rationale. Ann Intern Med 2002 May 21;136(10):760-764.

(20) National Institute for Health and Clinical Excellence. The care and management of osteoarthritis in adults. 2008;Clinical guidelines CG59.

(21) Pilling S, Anderson I, Goldberg D, Meader N, Taylor C, Two Guideline Development Groups. Depression in adults, including those with a chronic physical health problem: summary of NICE guidance. BMJ 2009 Oct 27;339:b4108.

(22) Mitchell AJ, Coyne JC. Do ultra-short screening instruments accurately detect depression in primary care? A pooled analysis and meta-analysis of 22 studies. Br J Gen Pract 2007 Feb;57(535):144-151.

(23) Mallen CD, Peat G. Screening older people with musculoskeletal pain for depressive symptoms in primary care. Br J Gen Pract 2008 Oct;58(555):688-693.

(24) Pignone MP, Gaynes BN, Rushton JL, Burchell CM, Orleans CT, Mulrow CD, et al. Screening for depression in adults: a summary of the evidence for the U.S. Preventive Services Task Force. Ann Intern Med 2002 May 21;136(10):765-776.

(25) Nisenson LG, Pepper CM, Schwenk TL, Coyne JC. The nature and prevalence of anxiety disorders in primary care. Gen Hosp Psychiatry 1998 Jan;20(1):21-28.

(26) Kroenke K, Spitzer RL, Williams JB, Monahan PO, Lowe B. Anxiety disorders in primary care: prevalence, impairment, comorbidity, and detection. Ann Intern Med 2007 Mar 6;146(5):317-325.

(27) Memel DS, Kirwan JR, Sharp DJ, Hehir M. General practitioners miss disability and anxiety as well as depression in their patients with osteoarthritis. Br J Gen Pract 2000 Aug;50(457):645-648.

(28) Hill S, Dziedzic K, Thomas E, Baker SR, Croft P. The illness perceptions associated with health and behavioural outcomes in people with musculoskeletal hand problems: findings from the North Staffordshire Osteoarthritis Project (NorStOP). Rheumatology (Oxford) 2007 Jun;46(6):944-951.

(29) Mallen CD. The prognosis of musculoskeletal pain in older adults in general practice. 2009.

(30) Addolorato G, Mirijello A, D'Angelo C, Leggio L, Ferrulli A, Abenavoli L, et al. State and trait anxiety and depression in patients affected by gastrointestinal diseases: psychometric evaluation of 1641 patients referred to an internal medicine outpatient setting. Int J Clin Pract 2008 Jul;62(7):1063-1069.

(31) Jackson JL, Passamonti M, Kroenke K. Outcome and impact of mental disorders in primary care at 5 years. Psychosom Med 2007 Apr;69(3):270-276.

(32) Creamer P, Lethbridge-Cejku M, Costa P, Tobin JD, Herbst JH, Hochberg MC. The relationship of anxiety and depression with self-reported knee pain in the community: data from the Baltimore Longitudinal Study of Aging. Arthritis Care Res 1999 Feb;12(1):3-7.

(33) Lang AJ, Stein MB. Screening for anxiety in primary care: why bother? Gen Hosp Psychiatry 2002 Nov-Dec;24(6):365-366.

(34) Katon W, Roy-Byrne P. Anxiety disorders: efficient screening is the first step in improving outcomes. Ann Intern Med 2007 Mar 6;146(5):390-392.

(35) Gilbody S, Sheldon T, House A. Screening and case-finding instruments for depression: a meta-analysis. CMAJ 2008 Apr 8;178(8):997-1003.

(36) Gilbody SM, House AO, Sheldon TA. Routinely administered questionnaires for depression and anxiety: systematic review. BMJ 2001 Feb 17;322(7283):406-409.

(37) Kamerow D. Depressed about depression. BMJ 2010 Oct 6;341:c5516.

(38) NHS Employers and the General Practitioners Committee. Quality and Outcomes Framework guidance for GMS contract 2009/10. Delivering investment in general practice. 2009.

(39) Eldridge SM, Ashby D, Feder GS. Informed patient consent to participation in cluster randomized trials: an empirical exploration of trials in primary care. Clin Trials 2005;2(2):91-98.

(40) Eldridge S, Kerry S, Torgerson DJ. Bias in identifying and recruiting participants in cluster randomised trials: what can be done? BMJ 2009 Oct 9;339:b4006.

(41) Carter BR, Hood K. Balance algorithm for cluster randomized trials. BMC Med Res Methodol 2008 Oct 9;8:65.

(42) National Institute for Health and Clinical Excellence. Generalised anxiety disorder and panic disorder (with or without agoraphobia) in adults. Management in primary, secondary and community care. 2011;Clinical guidelines CG113.

(43) Torgerson DJ. Contamination in trials: is cluster randomisation the answer? BMJ 2001 Feb 10;322(7282):355-357.

(44) Edwards SJ, Braunholtz DA, Lilford RJ, Stevens AJ. Ethical issues in the design and conduct of cluster randomised controlled trials. BMJ 1999 May 22;318(7195):1407-1409.

(45) Hutton JL. Are distinctive ethical principles required for cluster randomized controlled trials? Stat Med 2001 Feb 15;20(3):473-488.

(46) Kroenke K, Spitzer RL, Williams JB. The Patient Health Questionnaire-2: validity of a two-item depression screener. Med Care 2003 Nov;41(11):1284-1292.

(47) Lowe B, Wahl I, Rose M, Spitzer C, Glaesmer H, Wingenfeld K, et al. A 4-item measure of depression and anxiety: validation and standardization of the Patient Health Questionnaire-4 (PHQ-4) in the general population. J Affect Disord 2010 Apr;122(1-2):86-95.

(48) Von Korff M, Ormel J, Keefe FJ, Dworkin SF. Grading the severity of chronic pain. Pain 1992 Aug;50(2):133-149.

(49) Ware J,Jr, Kosinski M, Keller SD. A 12-Item Short-Form Health Survey: construction of scales and preliminary tests of reliability and validity. Med Care 1996 Mar;34(3):220-233.

(50) Tubach F, Baron G, Falissard B, Logeart I, Dougados M, Bellamy N, et al. Using patients' and rheumatologists' opinions to specify a short form of the WOMAC function subscale. Ann Rheum Dis 2005 Jan;64(1):75-79.

(51) Kroenke K, Spitzer RL, Williams JB. The PHQ-9: validity of a brief depression severity measure. J Gen Intern Med 2001 Sep;16(9):606-613.

(52) Lowe B, Unutzer J, Callahan CM, Perkins AJ, Kroenke K. Monitoring depression treatment outcomes with the patient health questionnaire-9. Med Care 2004 Dec;42(12):1194-1201.

(53) Spitzer RL, Kroenke K, Williams JB, Lowe B. A brief measure for assessing generalized anxiety disorder: the GAD-7. Arch Intern Med 2006 May 22;166(10):1092-1097.

(54) Sullivan MJL, Bishop SR. The Pain Catastrophizing Scale: Development and Validation. Psychological Assessment 1995;7(4):524.

(55) Brooks R. EuroQol: the current state of play. Health Policy 1996 Jul;37(1):53-72.

(56) de Vet HC, Heymans MW, Dunn KM, Pope DP, van der Beek AJ, Macfarlane GJ, et al. Episodes of low back pain: a proposal for uniform definitions to be used in research. Spine (Phila Pa 1976) 2002 Nov 1;27(21):2409-2416.

(57) Hunt IM, Silman AJ, Benjamin S, McBeth J, Macfarlane GJ. The prevalence and associated features of chronic widespread pain in the community using the 'Manchester' definition of chronic widespread pain. Rheumatology (Oxford) 1999 Mar;38(3):275-279.

(58) Lacey RJ, Lewis M, Jordan K, Jinks C, Sim J. Interrater reliability of scoring of pain drawings in a self-report health survey. Spine (Phila Pa 1976) 2005 Aug 15;30(16):E455-8.

(59) Kroenke K, Spitzer RL. The PHQ-9: A new depression diagnostic and severity measure. Psychiatr Ann 2002 SEP;32(9):509-515.

(60) Office for National Statistics. Standard Occupational Classification. 2002;Vol. 2. The coding Index.

(61) Office for National Statistics. The National Statistics Socio-economic classification user manual. 2002.

(62) Krumholz HM, Butler J, Miller J, Vaccarino V, Williams CS, Mendes de Leon CF, et al. Prognostic importance of emotional support for elderly patients hospitalized with heart failure. Circulation 1998 Mar 17;97(10):958-964.

(63) Diggle PJ, Heagerty P, Liang K-Y., Zeger SL. Analysis of Longitudinal Data. 2nd Edition ed. Oxford: Oxford university Press; 2002.

(64) National Institute for Health and Clinical Excellence. Guide to the Methods of Technology Appraisal. .

(65) Coventry PA, Hays R, Dickens C, Bundy C, Garrett C, Cherrington A, et al. Talking about depression: a qualitative study of barriers to managing depression in people with long term conditions in primary care. BMC Fam Pract 2011 Mar 22;12:10.

(66) Bryman A. Social Research Methods. 2nd ed. New York: Oxford University Press; 2004.

(67) Creswell J, Clark V. Designing and conducting mixed methods research. 2nd ed.: Sage; 2010.

**Appendix 1: Screening Template**

## Intervention arm

1. During the last month have you often been bothered by feeling down, depressed or hopeless?

Response: Yes / No

## During the last month have you often been bothered by little interest or pleasure in doing things?

## Response: Yes / No

1. During the last month have you often been bothered by feeling nervous, anxious or on the edge?

Response: Yes / No

1. During the last month have you often been bothered by not being able to stop or control worrying?

Response: Yes / No

1. How would you rate your pain on a 0-10 scale at the present time where 0 is ‘no pain’ and 10 is ‘pain as bad as could be’?

Response: 0-10

## Control arm

1. How would you rate your pain on a 0-10 scale at the present time where 0 is ‘no pain’ and 10 is ‘pain as bad as could be’?

Response: 0-10
